# Synthesis and Herbicidal Activity Against Buffelgrass (*Cenchrus ciliaris*) of (±)-3-deoxyradicinin

**DOI:** 10.3390/molecules24173193

**Published:** 2019-09-03

**Authors:** Giulia Marsico, Maria Sabrina Ciccone, Marco Masi, Fabrizio Freda, Massimo Cristofaro, Antonio Evidente, Stefano Superchi, Patrizia Scafato

**Affiliations:** 1 Department of Sciences, University of Basilicata, Via dell′Ateneo Lucano 10, 85100 Potenza, Italy; 2 Department of Chemical Sciences, University of Naples Federico II, Complesso Universitario Monte S. Angelo, Via Cintia 4, 80126 Napoli, Italy; 3 BBCA onlus, Via A. Signorelli 105, 00123 Rome, Italy; 4 ENEA C.R. Casaccia, SSPT-BIOAG-PROBIO, Via Anguillarese 301, 00123 Rome, Italy

**Keywords:** buffelgrass, phytotoxins, bioherbicides, radicinin, (±)-3-deoxyradicinin

## Abstract

A novel synthetic strategy for obtainment of (±)-3-deoxyradicinin (**2**) is reported. This synthetic methodology is more efficient than those previously reported in the literature and also shows higher versatility towards the introduction of different side-chains at both C-7 and C-2. The obtained compound (±)-**2** shows phytotoxicity against the grass-weed buffelgrass comparable to that of the natural phytotoxin radicinin (**1**). Therefore, (±)-**2** can constitute a more practical synthetic alternative to **1** as bioherbicide for buffelgrass control.

## 1. Introduction

**Figure 1:**
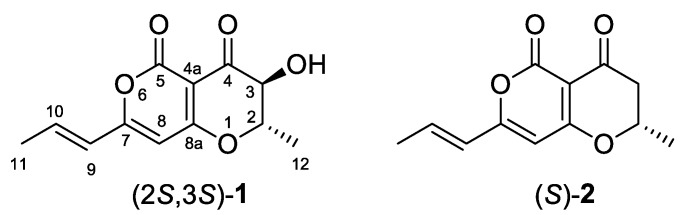
Structure of radicinin (**1**) and 3-deoxyradicinin (**2**).

Fungal phytotoxins constitute an essentially endless source of bioactive metabolites. Their large structural diversity reflects in a wide variety of bioactivities and potential applications in both medicine and agriculture. One of the most appealing application of fungal phytotoxins is in the development of bioherbicides, which show lower or nil toxicity, and then a lower environmental and ecological impact, than the traditional synthetic pesticides [1,2,3]. For this reason, these are now emerging as a very promising tool for weed management. The search for new and selective bioherbicides usually starts with the isolation of phytotoxins from weed pathogens [3]. This strategy has been followed also for the search of selective compounds for the control of buffelgrass (*Pennisetum ciliare* or *Cenchrus ciliaris*), a perennial grass-weed native to Africa, the Mediterranean area, and Middle-East [4], which has become highly invasive in North America and Australia where it is a menace for the native vegetation, infesting roadsides and urban landscapes and promoting wildfires. Buffelgrass control is thus an emerging concern in those countries, where it is now carried out by broad-spectrum herbicides which, however, can heavily damage non-target native plants [5]. Looking for novel bioherbicides for this weed control, the production of phytotoxic metabolites by the buffelgrass foliar pathogens *Cochliobolus australiensis* and *Pyricularia grisea* has been recently investigated [6,7,8]. From the liquid culture of *C. australiensis*, the known phytotoxin radicinin (**1**) (Figure 1) and a new dihydropyran-pyran-4,5-dione named cochliotoxin were isolated together with their close metabolites 3-*epi*-radicinin, radicinol and 3-*epi*-radicinol [6]. Radicinin (**1**) was first isolated from the fungus *Stemphylium radicinum* [9] and its structure was elucidated some years later by chemical and spectroscopic studies [10] while the absolute configuration was established by X-ray analysis of its 4-*p*-bromobenzoate [11]. Compound **1** has been also found to be produced by many other fungal species [11,12,13,14,15,16,17]. Phytotoxins **1** and cochliotoxin, together with the other compounds isolated from *P. grisea* [8], were assayed for their phytotoxic activity on host and non-host indigenous plants. Radicinin (**1**) demonstrated high target-specific toxicity on buffelgrass, low toxicity to native plants, and no teratogenic, sub-lethal, or lethal effects on zebrafish (*Brachydanio rerio*) embryos [18]. Previous studies had also shown its antifungal, insecticidal and plant growth regulatory activity, as well as antibiotic activity against Gram-positive bacteria, including *Staphylococcus aureus* [19,20]. More recently, the ability of **1** to inhibit *Xylella fastidiosa*, the causal agent of many devastating plant diseases, including Pierce′s Disease of grapevine, phony peach disease, alfalfa dwarf disease, plum leaf scald, citrus variegated chlorosis, and leaf scorch of almond, coffee, elm, oak, oleander, pear, and sycamore was reported [21]. Although the high and specific bioactivity of **1** against buffelgrass makes this phytotoxin very promising for the development of bioherbicides for the control of this weed, the low quantity of **1** available from fungal sources constitute a real bottleneck for such development, do not allowing open field studies [22]. Furthermore, the large scale production of fungal natural products requires the use of fermenters where the sticking of fungal mycelium to walls and blades often results as the most critical problem, strongly affecting the production yield [23]. For these reasons, we considered worthwhile to develop an efficient synthetic strategy for the obtainment of **1** or some its active analogues. In particular, we faced the synthesis of racemic (±)-3-deoxyradicinin (**2**) (Figure 1) the immediate biosynthetic precursor of **1** [24] with the aim to define the effect on phytotoxicity of both the absolute configuration and the presence of the hydroxy moiety at C-3. In fact, a recent structure-activity relationship study carried out on **1** by some of us demonstrated that the presence of an α,β-unsaturated carbonyl group at C-4 is essential for the phytotoxic activity and that the double bond of the side chain at C-7 also play a role to impart activity [25]. However, the effect on phytotoxicity of the hydroxy group at C-3 was not ascertained with certainty. The absolute configuration at C-3 seemed to have some role, because 3-*epi*-radicinin was found to be less active, but the effect of the absolute stereochemistry at C-2 was not investigated [25].

Our aim was then to develop an efficient synthetic strategy to obtain (±)-**2** in high yield, starting from commercially available products. The synthetic strategy was also designed to be versatile for the synthesis of analogues of **2** with different side-chains at C-7. Moreover, the transformation of **2** to **1** has been reported in the literature [26], therefore, a novel approach to (±)-**2**, will also constitute a formal synthesis of (±)-**1**.

## 2. Results and Discussion

### 2.1. Total Synthesis of (±)-3-deoxyradicinin (***2***)

To the best of our knowledge, only two approaches to total synthesis of (±)-**2** have been reported in the literature so far. However, the first procedure, reported by Kato et al. in 1969 [26], appears unpractical, providing (±)-**2** in five steps and <10% overall yield, but from not commercially available precursors which, in turn, require some additional steps for preparation [27]. On the other hand, the second one by Suzuki et al. [24], although leading to (±)-**2** in only four steps from triacetic lactone methyl ether, still provides only 1% of overall yield. In addition, both approaches appear to be poorly versatile to other pyrones differently substituted at C-7.

**Scheme 1:**
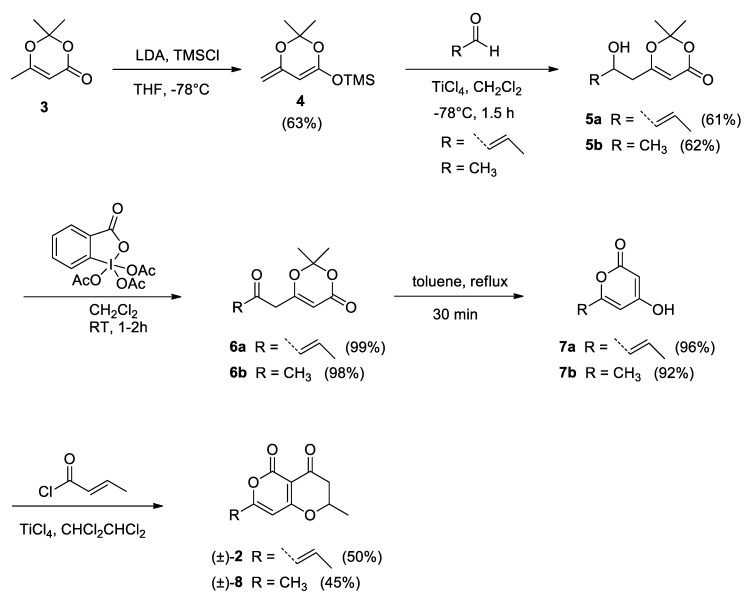
Synthesis of (±)-3-deoxyradicinin (**2**) and analogue (±)-**8**.

We then decided to apply a different approach, starting from commercially available 2,2,6-trimethyl-4*H*-1,3-dioxin-4-one (**3**) (Scheme 1).

Accordingly, the dioxinone **3** was enolized with lithium diisopropylamide (LDA) at −78 °C in THF and trapped with trimethylsilyl chloride (TMSCl), providing the silyl dienolether **4** in 63% yield after high vacuum distillation (pressure lower than 1.0 mmHg) of the crude product. This yield may be considered satisfactory, taking into account that the purification of **4** by distillation shall be carried out on strictly controlled temperature conditions because it decomposes when heated at temperatures higher than 50 °C. The silyl enol ether **4** was reacted with crotonaldehyde in Mukaiyama conditions in the presence of TiCl_4_ in dichloromethane at −78 °C, affording the alcohol **5a** in 61% yield. Oxidation of **5a** with Dess-Martin periodinane gave rise to the corresponding ketone **6a** in practically quantitative yield. The latter compound spontaneously eliminated acetone and cyclized upon heating in toluene, providing the hydroxy α-pyrone **7a** in 96% yield. Finally, acylation of **7a** with crotonyl chloride, followed by *in situ* intramolecular Michael addition of the hydroxy moiety, provided the desired deoxyradicinin (±)-**2** in 50% yield (overall yield 18%). Notably, such synthetic strategy is versatile towards the synthesis of radicinin analogues with different substituents at C-7 and C-2. In fact, by simply employing a different aldehyde in the Mukaiyama reaction with compound **4** can be introduced a different side-chain at C-7, while the use of a different α,β-unsaturated acid chloride in the last step allows to insert a different substituent at C-2. This potentiality was verified by the synthesis of the methyl-substituted dihydropyran-pyran-4,5-dione (±)-**8** which was obtained following the same synthetic procedure described for (±)-**2** (Scheme 1). In fact, the Mukaiyama reaction of **4** with acetaldehyde provided alcohol **5b** which, after oxidation to **6b** and subsequent cyclization, provided the pyrone **7b**. The acylation with crotonyl chloride finally provided (±)-**8** in similar (17%) overall yield.

**Scheme 2:**
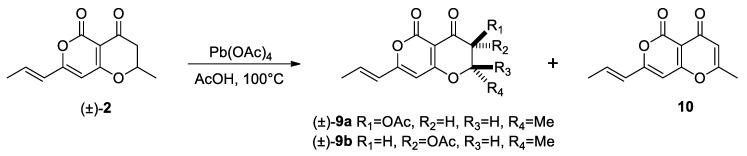
Acetoxylation of (±)-3-deoxyradicinin (**2**) with Pb(OAc)_4_ (bold and dashed lines in figure refer to the relative configuration).

With (±)-**2** in hand, we considered worthwhile to try its conversion to (±)-**1** following the oxyacetylation procedure described in the literature by Kato et al. [26] (Scheme 2). Accordingly, (±)-**2** was treated with lead tetracetate in acetic acid, stirring at 100 °C for 2h. Contrary to what reported in the literature, where obtainment of only 3-acetoxy radicinin was claimed, we got 6:4 *trans*:*cis* mixture of (±)-radicinin acetate **9a** and 3-*epi*-(±)-radicinin acetate **9b** (19% overall) together with the unsaturated pyranopyrandione **10** (74%) as the main product of the reaction. The latter probably formed from (±)-**9** through spontaneous acetic acid elimination. The low yield obtained, the low diastereoselectivity observed and, most importantly, the large formation of **10** as a by-product in the present reaction makes necessary a further optimization of such synthetic transformation, possibly trying different reagents for the α-oxidation of 3-deoxyradicinin (**2**). This type of study is out of the scope of the present research and will be developed successively. However, the availability of the radicinin derivative **10** prompted us to test also this compound in the phytotoxicity assays on *C. ciliaris* (*vide infra*).

### 2.2. Phytotoxicity Assays on C. ciliaris

**Figure 2:**
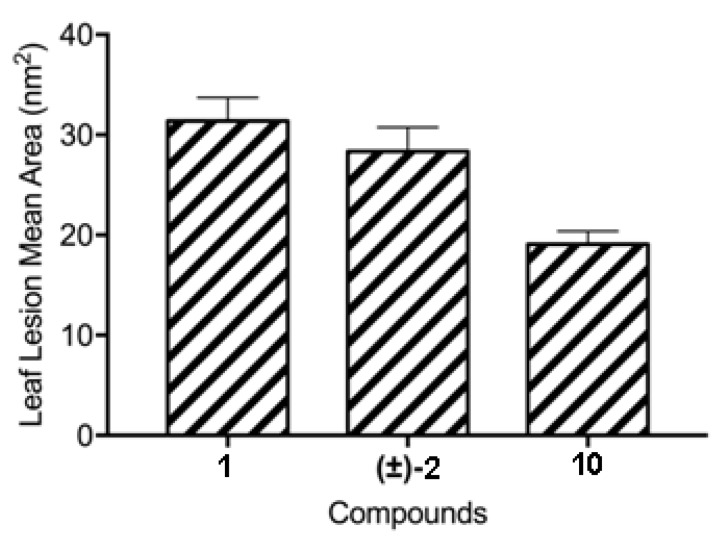
Results of leaf puncture bioassay on buffelgrass (*C. ciliaris*) at concentration of 2.5 × 10^−3^ M for the synthetic derivatives (±)-**2** and **10** and the natural compound **1**.

Compounds (±)-**2** and **10** were tested by buffelgrass (*C. ciliaris*) leaf puncture assay at 2.5 × 10^−3^ M as reported in Materials and Methods section and their activity was evaluated in comparison with that showed by the natural metabolite radicinin (**1**) previously reported by Masi et al. [18] (Figure 2).

As inferred from Figure 2, the activity of compound (±)-**2** is comparable to that of the natural radicinin (2*S*,3*S*)-**1**, while the activity of the pyranopyrandione **10** is slightly lower. The lack of the hydroxy group on C-3 in **2** seems to suggest, contrary to previous observations [25], that this moiety is not essential for the compounds phytotoxicity. Moreover, the comparable activity of racemic **2** and non-chiral compound **10** with optically active (2*S*,3*S*)-**1**, highlight that the absolute configuration of the methyl group at C-2 also has a minor influence on phytotoxicity. As far as absolute configuration at C-3 is concerned, previous results have shown that in the presence of a hydroxy moiety on this stereocenter its stereochemistry results very important to impart a strong activity considering the significant reduction in phytotoxicity showed by (2*S*,3*R*)-3-*epi*-radicinin in comparison with (2*S*,3*S*)-**1** [25].

The obtained results highlight that derivatives like (±)-**2** and **10**, much simpler to be prepared than optically active radicinin (**1**) can be considered as valid alternative to this phytotoxin as possible bioherbicides against buffelgrass. Moreover, the possibility to employ, with similar results, racemic or non-chiral compounds in place of enantiopure ones is a great advantage for the preparation of the large amounts of compounds necessary for the field test of bioherbicides. In fact, it is obviously much simpler to prepare on large scale a racemic compound than an optically active one, which usually require to employ expensive chiral reagents or catalysts.

## 3. Materials and Methods 

### 3.1. General Experimental Procedures

^1^H (400 MHz) and ^13^C (100 MHz) NMR spectra were recorded in CDCl_3_ or DMSO-d6 on a Varian INOVA 400 spectrometer, using TMS as an internal standard (see Supplementary Materials). GC/MS spectra were recorded on a HP 6890 gas chromatograph, equipped with a HP-5975 mass spectrometric detector. HRESI mass spectra were recorded on Agilent Technologies 1100 LC/MS TOF instrument. Analytical TLC was performed on silica gel 60 Macherey–Nagel sheets. The spots were visualized by exposure to UV radiation (254 nm) and/or by spraying the plates with a potassium permanganate solution. Column chromatography was performed using silica gel (Merck, Kieselgel 60, (60–230 mesh). THF was freshly distilled before its use on sodium benzophenone ketyl under nitrogen atmosphere. Diisopropylamine (DIPA) and CH_2_Cl_2_ were dried by distillation over calcium hydride and stored under a nitrogen atmosphere. Trimethylsilyl chloride (TMSCl), crotonaldehyde, and crotonoyl chloride were distilled and stored under argon atmosphere before their use. Commercially available *n*-butyllithium (Aldrich) was a 2.5 M solution in hexanes. The other analytical grade solvents and commercially available reagents were used without further purification.

### 3.2. ((2,2-Dimethyl-4-methylene-4H-1,3-dioxin-6-yl)oxy)-trimethylsilane (**4**)

A solution of DIPA (5.4 mL, 1.1 eq) in THF was stirred under nitrogen atmosphere and cooled to 0 °C, then *n*-butyllithium 2.5 M (15.4 mL, 1.1 eq) was slowly added in about 10 min and the mixture was stirred at the same temperature for another 45 min. Afterwards, the pale yellow solution was cooled to −78 °C and 2,2,6-trimethyl-4*H*-1,3-dioxin-4-one **3** (4.65 mL, 35 mmol) was added in 5 min. After 1h, also freshly distilled TMSCl (5.33 mL, 1.2 eq) was added in about 10 min; the dark orange mixture was stirred for 1 h and then slowly heated to room temperature. The mixture was filtered under reduced pressure, the solid was washed with hexanes and the filtrate concentrated by distillation under a reduced pressure. The crude product was purified by distillation under a high vacuum, using a rotary pump (pressure less than 1 mmHg) and heating to 50°C. The pure silyl dienolether **4** [28] (4.76 g, 63%) corresponded to the collected fraction having *bp* = 32–34 °C.

^1^H-NMR (400 MHz, CDCl_3_): δ 0.27 (s, 9H, Si(C*H*_3_)_3_), 1.55 (s, 6H, C(C*H*_3_)_2_), 3.88 (s, 1H, C=C*Ha*H), 4.07 (s, 1H, C=H*Hb*), 4.65 (s, 1H, C=C*H*). ^13^C-NMR (100 MHz, CDCl_3_): δ 0.24, 24.5, 76.6, 84.9, 102.5, 151.8, 153.3.

### 3.3. (E)-6-(2-hydroxypent-3-en-1-yl)-2,2-dimethyl-4H-1,3-dioxyn-4-one (**5a**)

A solution of crotonaldehyde (2.02 mL, 24.4 mmol, 1.1 eq) in 90 mL of CH_2_Cl_2_ was cooled to −78 °C under a nitrogen atmosphere, TiCl_4_ (2.43 mL, 22.2 mmol, 1.0 eq) and silyl dienolether **4** (4.76 g, 22.2 mmol) was slowly added in sequence. After 1.5 h of stirring at the same temperature, the mixture was quenched with 40 mL of a saturated solution of NaHCO_3_ and the organic phase separated. The aqueous phase was again extracted with CH_2_Cl_2_, the combined organic phases were dried over anhydrous sodium sulfate and the solvent eliminated under reduced pressure. The crude product was purified by column chromatography on silica gel (first *n*-hexane:ethyl acetate 7:3 and then *n*-hexane:EtOAc 1:1), obtaining pure alcohol **5**a [29] (2.87 g) in 61% yield.

^1^H-NMR (400 MHz, CDCl_3_): δ 1.69 (s, 3H, CC*H*_3_CH_3_), 1.70 (s, 3H, CCH_3_C*H*_3_), 1.71 (d, *J* = 7.2 Hz, 3H, CH=CHC*H*_3_), 2.40-2.48 (m, 2H, C*H*_2_CHOH), 4.36 (bq, *J* = 6.4 Hz, 1H, C*H*OH), 5.32 (s, 1H, C=C*H*), 5.51 (dd, *J* = 15.2 Hz, 7.2 Hz, 1H, C*H*=CHCH_3_), 5.71–5.78 (m, 1H, CH=C*H*CH_3_). ^13^C-NMR (100 MHz, CDCl_3_): δ 17.6, 24.9, 25.3, 41.5, 69.8, 95.2, 106.6, 128.5, 132.3, 161.1, 168.5.

### 3.4. 6-(2-Hydroxypropyl)-2,2-dimethyl-4H-1,3-dioxyn-4-one (**5b**)

Following the same procedure as above, but starting from freshly distilled acetaldehyde, alcohol **5b** [30] was obtained in 62% yield.

^1^H-NMR (400 MHz, CDCl_3_): δ 1.27 (d, 3H, *J* = 6.0 Hz, CH-C*H*_3_), 1.70 (s, 6H, C(C*H*_3_)_2_), 1.86 (br. s, 1H, O*H*), 2.38 (d, 2H, *J* = 6.4 Hz, CH-C*H*_2_), 4.09–4.13 (m, 1H, C*H*-CH_2_), 5.33 (s, 1H, C=C*H*). ^13^C-NMR (100 MHz, CDCl_3_): δ 23.5, 24.8, 25.2, 43.2, 65.2, 95.0, 106.6, 161.3, 169.0.

### 3.5. (E)-2,2-dimethyl-6-(2-oxopent-3-en-1-yl)-4H-1,3-dioxyn-4-one (**6a**)

Dess-Martin periodinane (2.50 g, 1.3 eq) was added in small portions to a solution of alcohol **5a** (940 mg, 4.43 mmol) in 20 mL of CH_2_Cl_2_ and the solution was stirred at room temperature, monitoring the conversion by TLC analysis. After 1.5 h, the mixture was diluted with 120 mL of diethyl ether and 24 mL of a saturated solution of sodium bicarbonate containing 5% sodium thiosulfate and stirred for further 20 min. The organic layer was separated, dried over anhydrous sodium sulfate and then concentrated under reduced pressure. The crude product was purified by column chromatography on silica gel (first *n*-hexane:EtOAc 7:3 and then *n*-hexane:EtOAc 1:1), to afford pure ketone **6a** [29] (912 mg, 98% yield).

^1^H-NMR (400 MHz, CDCl_3_): δ 1.71 (s, 6H, C(C*H*_3_)_2_), 1.95 (d, *J* = 6.8 Hz, 3H, CH=CHC*H*_3_), 3.46 (s, 2H, COC*H*_2_), 5.36 (s, 1H, C=C*H*), 6.17 (d, *J* = 15.6 Hz, 1H, C*H*=CHCH_3_), 6.90–6.97 (m, 1H, CH=C*H*CH_3_). ^13^C-NMR (100 MHz, CDCl_3_): δ 18.4, 25.0, 44.6, 96.6, 107.1, 130.8, 145.4, 160.7, 165.0, 192.3.

### 3.6. 2,2-Dimethyl-6-(2-oxopropyl)-4H-1,3-dioxyn-4-one (**6b**)

Following the same procedure as above ketone **6b**, [30] was obtained in 98% yield.

^1^H-NMR (400 MHz, CDCl_3_): δ 1.72 (s, 6H, C(C*H*_3_)_2_), 2.25 (s, 3H, CO-C*H*_3_), 3.35 (s, 2H, CO-C*H*_2_), 5.36 (s, 1H, C=C*H*). ^13^C-NMR (100 MHz, CDCl_3_): δ 25.0, 30.1, 47.9, 96.7, 107.2, 160.6, 164.3, 184.3.

### 3.7. (E)-4-hydroxy-6-(propen-1-yl)-2H-piran-2-one (**7a**)

Ketone **6a** (912 mg, 4.34 mmol) was dissolved in 40 mL of toluene and the solution was heated to 100 °C for 0.5–1 h. During this time, a very thin white precipitate was formed. After cooling to room temperature, toluene was removed at reduced pressure and the solid was washed with diethyl ether and a small amount of chloroform. ^1^H-NMR analysis in DMSO-d6, being it insoluble in chloroform, confirmed it as pure pyranodienone **7a** [29] (632 mg, 96% yield).

^1^H-NMR (400 MHz, DMSO-d_6_) δ 1.82 (d, *J* = 6.4 Hz, 3H, CH=CHC*H*_3_), 5.22 (s, 1H, C=C*H*), 5.98 (s, 1H, C=C*H*), 6.15 (d, *J* = 16.8 Hz, 1H, C*H*=CHCH_3_), 6.41–6.49 (m, 1H, CH=C*H*CH_3_), 11.63 (s, 1H, O*H*). ^13^C NMR (100 MHz, DMSO-d_6_) δ 23.2, 94.6, 104.9, 128.5, 138.9, 164.1, 168.1, 175.5. MS(EI): *m*/*z* 152 (51, M^+^), 137 (50), 124 (34), 69 (100), 55 (11).

### 3.8. 4-Hydroxy-6-(methyl)-2H-piran-2-one (**7b**)

Following the same procedure as above, 4-hydroxypyranone **7b** [31] was obtained in 92% yield.

^1^H-NMR (400 MHz, DMSO-d_6_): δ 2.12 (s, 3H, C=C-C*H*_3_), 5.17 (s, 1H, C=C*H*), 5.92 (s, 1H, CO-C*H*), 11.58 (s, 1H, O*H*). ^13^C-NMR (100 MHz, DMSO-d_6_): δ 35.9, 93.3, 105.4, 168.5, 169.1, 175.7.

### 3.9. (E)-2-methyl-7-(prop-1-en-1-yl)-2,3-dihydropyrane[4,3-b]pyran-4,5-dione ((±)-3-deoxyradicinin; (±)-**2**)

Under a nitrogen atmosphere, TiCl_4_ (0.234 mL, 213 mmol, 1.8 eq) and crotonoyl chloride (0.170 mL, 1.77 mmol, 1.5 eq) were dropwise added in sequence to a solution of pyranodienone **7a** (180 mg, 1.18 mmol) in 2 mL of CHCl_2_CHCl_2_. The mixture was first stirred for 15 min at room temperature and then heated to 100 °C for 3 h. After cooling to room temperature, the mixture was poured into water/ice (10 mL) and extracted with ethyl acetate. The organic layer was washed with brine and concentrated under reduced pressure. The crude product was purified by column chromatography on silica gel (*n*-hexane:acetone 1:1), to afford (±)*-***2** [24] (130 mg) in 50% yield.

^1^H-NMR (400 MHz, CDCl_3_): δ 1.54 (d, *J* = 6.4 Hz, 3H, CHC*H*_3_), 1.95 (d, *J* = 6.8 Hz, 3H, CH=CHC*H*_3_), 2.59–2.70 (m, 2H, COC*H*_2_), 4.73–4.80 (m, 1H, OC*H*CH_3_), 5.83 (s, 1H, C=C*H*), 6.02 (d, *J* = 15.6 Hz, 1H, C*H*=CHCH_3_), 6.89–6.97 (m, 1H, CH=C*H*CH_3_). ^13^C-NMR (100 MHz, CDCl_3_): δ 18.7, 20.3, 43.7, 53.4, 98.1, 100.2, 122.7, 140.0, 157.2, 163.4, 175.9, 186.4. MS(EI): *m*/*z* 220 (44, M^+^), 205 (100), 179 (24), 111 (17), 69 (77). HRESIMS (+) *m*/*z* 221.0810 [M + H]^+^ (calcd for C_12_H_13_O_4_ 221.0814).

### 3.10. 2,7-Dimethyl-2,3-dihydropyrane[4,3-b]pyran-4,5-dione ((±)-**8**)

Following the same procedure as above, the compound analogue (±)-**8** [32] was obtained in 45% yield.

^1^H-NMR (400 MHz, CDCl_3_): δ 1.54 (d, *J* = 6.8 Hz, 3H, CH-C*H*_3_), 2.27 (s, 3H, CH=C-C*H*_3_), 2.59–2.68 (m, 2H, CH-C*H*_2_), 4.74–4.79 (m, 1H, C*H*-CH_2_), 5.90 (s, 1H, C=C*H*). ^13^C-NMR (100 MHz, CDCl_3_): δ 20.2, 20.7, 29.2, 43.7, 95.1, 99.6, 157.8, 168.7, 175.9, 186.4. MS(EI): *m*/*z* 194 (28, M^+^), 179 (100), 153 (16), 69 (61), 41 (16). HRESIMS (+) *m*/*z* 195.0651 [M + H]^+^ (calcd for C_10_H_11_O_4_ 195.0657).

### 3.11. Oxidation of (±)-3-deoxyradicinin (**2**) with Pb(OAc)_4_

A mixture of compound **2** (40 mg, 0.18 mmol), Pb(OAc)_4_ (120 mg, 1.5 eq) and acetic acid (3 mL) was stirred and heated to 100 °C for 2h. The mixture was then cooled to room temperature, poured into water/ice and extracted with dichloromethane. The organic layer was washed with a 5% NaHCO_3_ solution and brine and concentrated under reduced pressure. By column chromatography on silica gel (*n*-hexane: acetone 1:1) of the residue, two fractions were eluted, corresponding to a 6:4 mixture of *trans*:*cis* (±)-radicinin acetate **9** [21] (9.5 mg, 19%) and the pyranopyrandione **10** [26] (29 mg, 74%).

(*E*)-2-methyl-4,5-dioxo-7-(prop-1-en-1-yl)-2,3,4,5-tetrahydropyrano[4,3-b]pyran-3-yl acetate (**9a** + **9b** mixture). ^1^H-NMR (400 MHz, CDCl_3_): δ 1.45 (d, *J* = 6.4 Hz, 3H, CHC*H*_3_, **9b**), 1.55 (d, *J* = 6.4 Hz, 3H, CHC*H*_3_, **9a**), 1.97 (d, *J* = 7.2 Hz, 3H, CH=CHC*H*_3_, **9a** + **9b**), 2.17 (s, 3H, COC*H*_3_, **9b**), 2.21 (s, 3H, COC*H*_3_, **9a**), 4.69–4.75 (m, 1H, C*H*CH_3_, **9a**), 4.85–4.92 (m, 1H, C*H*CH_3_, **9b**), 5.25 (d, *J* = 10.8 Hz, 1H,C*H*OAc, **9a**), 5.49 (d, *J* = 3.6 Hz, 1H, C*H*OAc, **9b**), 5.85 (s, 1H, C=C*H*, **9a** + **9b**), 6.04 (dd, *J* = 15.2 Hz, 1.6 Hz, 1H, C*H*=CHCH_3_, **9a** + **9b**), 6.93–7.00 (m, 1H, CH=C*H*CH_3_, **9a** + **9b**).

(*E*)-2-methyl-7-(prop-1-en-1-yl)pyrano[4,3-b]pyran-4,5-dione (**10**). ^1^H-NMR (400 MHz, CDCl_3_): δ 1.97 (d, *J* = 7.2 Hz, 3H, CH=CHC*H*_3_), 2.30 (s, 3H, CH=CC*H*_3_), 6.06 (s, 1H, C*H*=CCH_3_), 6.07 (d, *J* = 15.6 Hz, 1H, C*H*=CHCH_3_), 6.17 (s, 1H, C=C*H*), 6.90–6.99 (m, 1H, C=C*H*-CH_3_). ^13^C-NMR (100 MHz, CDCl_3_): δ 18.7, 19.6, 96.7, 105.7, 115.7, 122.3, 139.8, 156.9, 162.2, 163.6, 169.3, 174.3. HRESIMS (+) *m*/*z* 219.0653 [M + H]^+^ (calcd for C_12_H_11_O_4_ 219.0657).

### 3.12. Leaf Puncture Bioassays

The synthetic derivatives (±)-**2** and **10** were assayed at 2.5 × 10^−3^ M for phytotoxicity on leaves of buffelgrass (*C. ciliaris*) following a procedure previously reported [22,25] and their activity compared with that of natural **1** [18]. Accordingly, compounds were first dissolved in MeOH (final concentration 4%) and stock solutions using sterile distilled water were then prepared. An incision of ca. 3 mm was made on the adaxial surface of each leaf section of 3 cm with an insulin needle. The leaf sections were placed in groups of six on the surface of a water-saturated filter paper in each of four petri dishes. Five leaf sections in each petri dish were tested with the solution containing the compound, while one leaf section was used as a negative control (4% MeOH only). A droplet (10 μL) of the appropriate solution was applied over each needle incision using a micropipette. The dishes were sealed with parafilm and incubated at 24 °C for 3 days in a temperature-regulated chamber under a photoperiod of 14–10 h (light/dark). After three days of treatment, necrotic lesion development was evaluated by removing the petri dish cover, placing a glass disc on the leaf sections to flatten them into a single plane, and photographing each dish with its leaf sections. Each acquired image was then analyzed with the software ImageJ to measure the necrotic area caused by the solution.

### 3.13. Statistical Analyses

Statistical analyses were carried out using the GraphPad Prism 8 software. Data were represented as the mean ± standard deviation and analyzed for statistical significance using ordinary one-way or two-ways analysis of variance (ANOVA) and multiple comparisons. For all test, *P* < 0.5 was considered to indicate a statistically significant difference.

## 4. Conclusions

A novel efficient synthetic strategy for obtainment of (±)-3-deoxyradicinin (**2**) has been developed. This synthetic methodology not only is more efficient than those previously reported in the literature, but it also shows higher versatility towards the introduction of different side-chains at both C-7 and C-2 as shown with the synthesis of the analogue (±)-**8**. The obtained compound (±)-**2** shows phytotoxicity against the grass-weed buffelgrass comparable to that of the natural phytotoxin radicinin (**1**). Therefore, (±)-**2** can constitute a more practical synthetic alternative to **1** as bioherbicide for buffelgrass control. In fact, while **1** is available only in very small amount by fungal culture, (±)-**2** can be obtained in much larger quantities by organic synthesis thus allowing to carry out extensive studies on its phytotoxic activity and even open field tests. These results are then of interest for both synthetic and natural products chemistry as well as for the nowadays forefront research on bioherbicides for weed control in agriculture.

## Supplementary Materials

The following are available online. ^1^H-NMR and ^13^C-NMR spectra of compounds (±)-**2**, **4**, **5a**,**b**, **6a**,**b**, **7a**,**b**, (±)-**8**, (±)-**9a**,**b**, **10**.
