# Supplementary material for: Synthesis and Herbicidal Activity Against Buffelgrass (Cenchrus ciliaris) of (±)-3-deoxyradicinin
Source: Molecules. 2019 Sep 3;24(17):3193. doi: 10.3390/molecules24173193 (PMC6749313; doi:10.3390/molecules24173193)

Supporting Information

Synthesis and herbicidal activity against buffelgrass (*Cenchrus ciliaris*) of (±)-3-deoxyradicinin

Giulia Marsico[1], Maria Sabrina Ciccone[1], Marco Masi[2,], Fabrizio Freda[3], Massimo Cristofaro[3,4], Antonio Evidente[2], Stefano Superchi[1,*], and Patrizia Scafato[1,*]

[1] *Department of Sciences, University of Basilicata, Via dell'Ateneo Lucano 10, 85100 Potenza, Italy;*
[2] *Department of Chemical Sciences, University of Naples Federico II, Complesso Universitario Monte S. Angelo, Via Cintia 4, 80126 Napoli, Italy;*
[3] *BBCA onlus, Via A. Signorelli 105, 00123 Rome, Italy;*
[4] *ENEA C.R. Casaccia, SSPT-BIOAG-PROBIO, Via Anguillarese 301, 00123 Rome, Italy.*

Figure S1. ^{1}HNMR spectrum of compound **4**

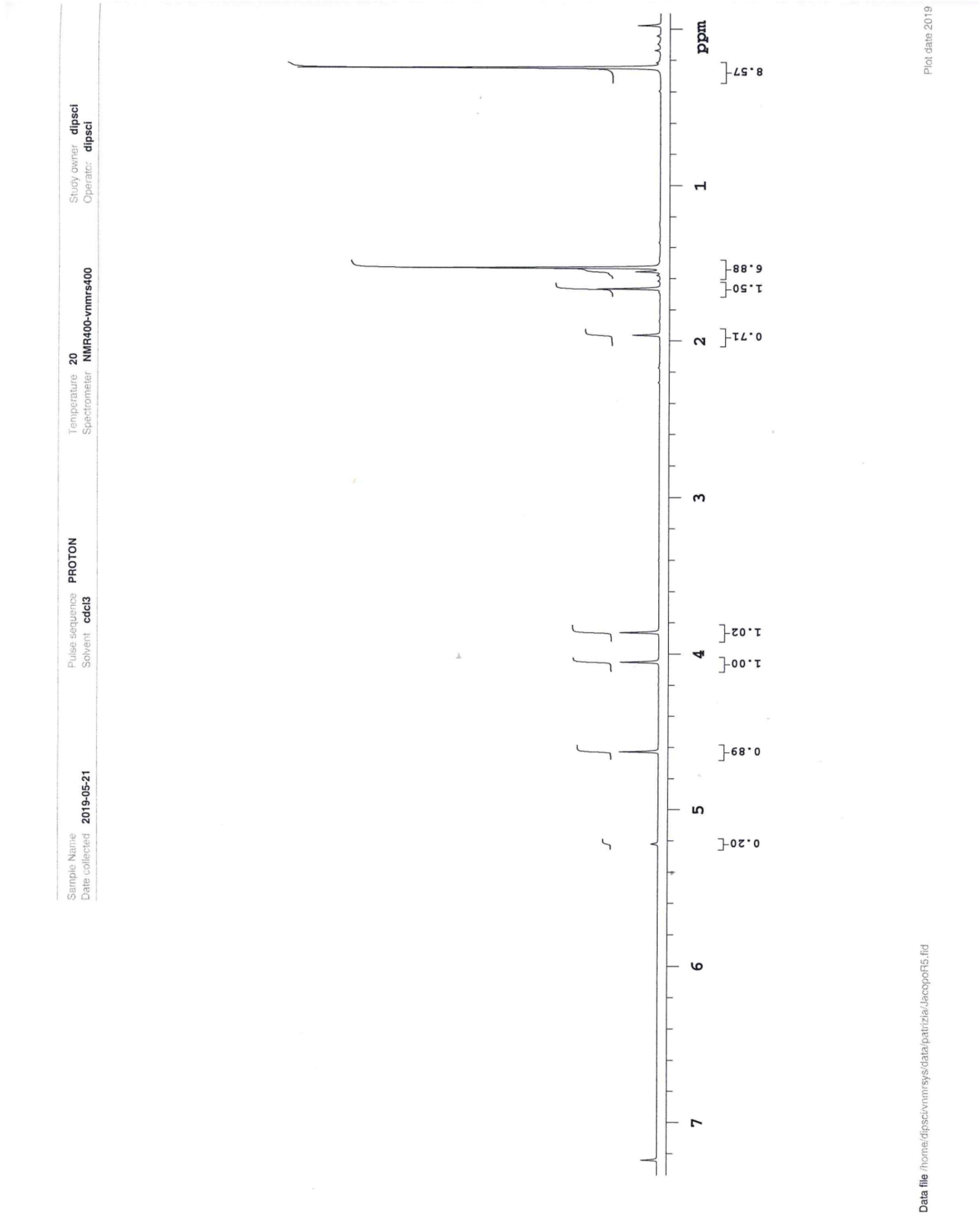

Figure S2. ^{13}CNMR spectrum of compound **4**

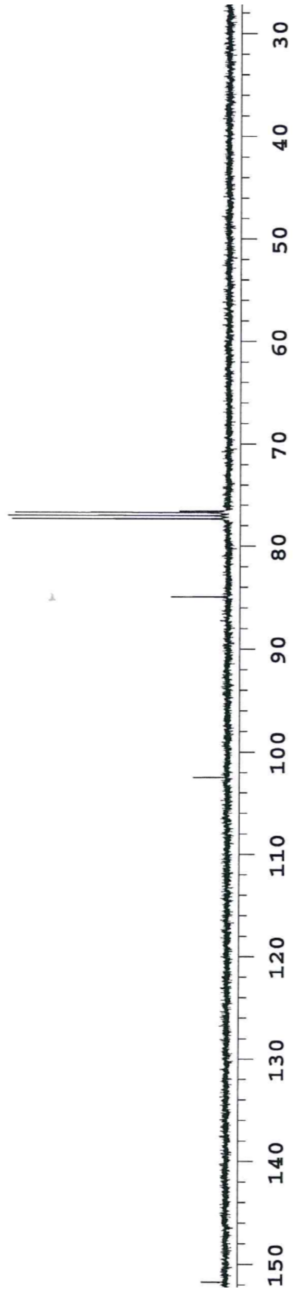

Figure S3. ^{1}HNMR spectrum of compound **5a**

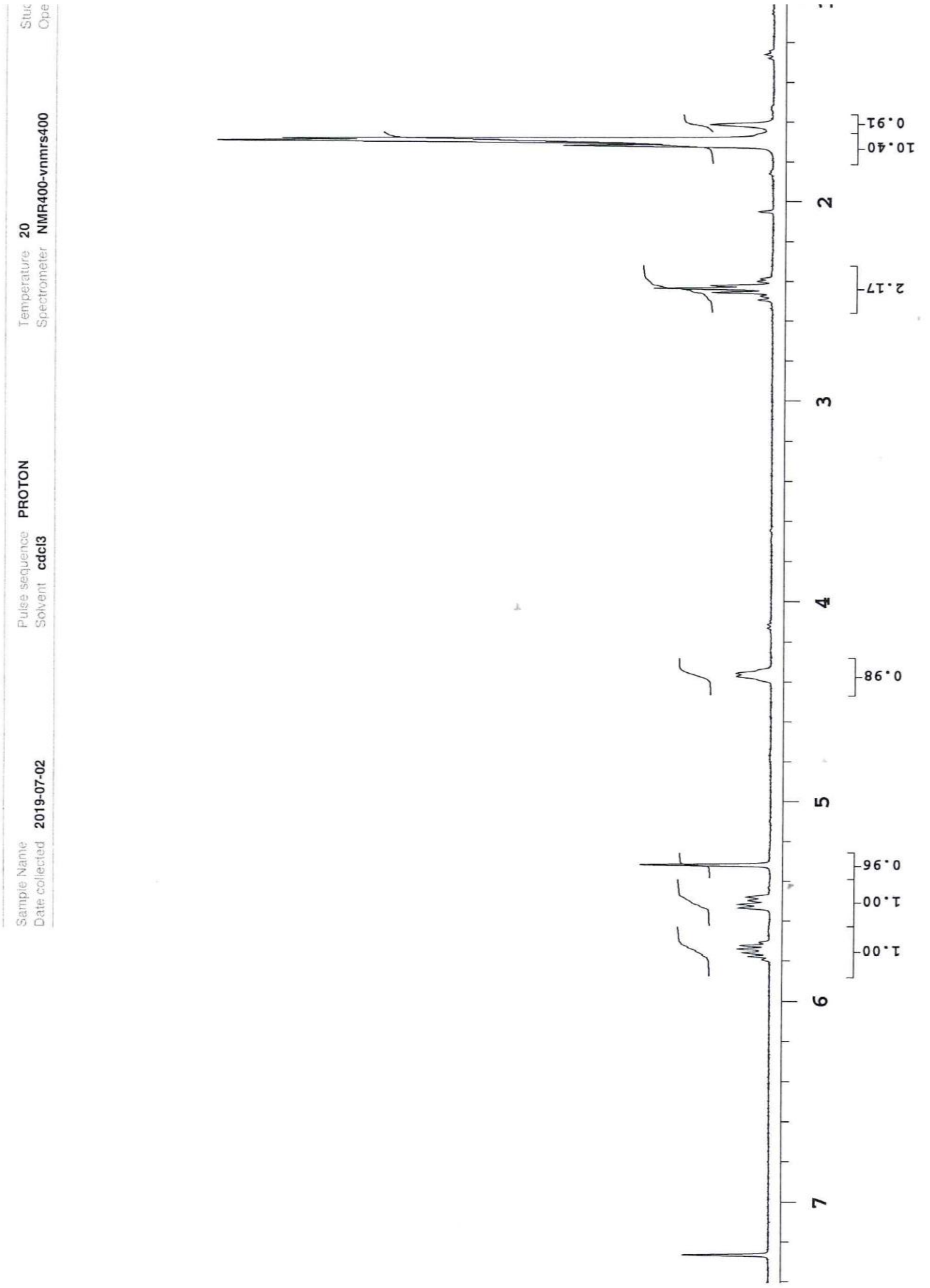

Figure S4. ^{13}CNMR spectrum of compound **5a**

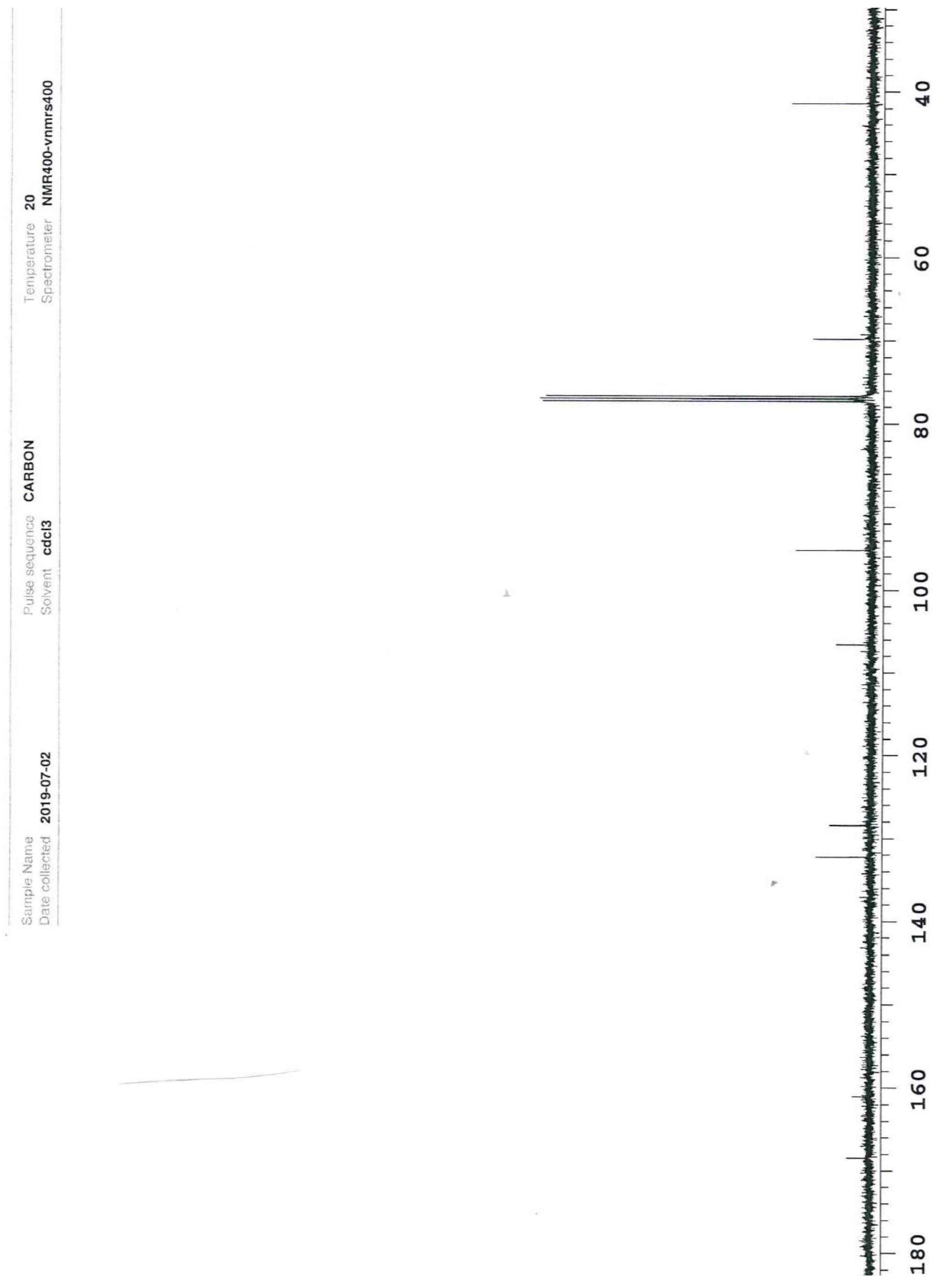

Figure S5. ^{1}HNMR spectrum of compound **6a**

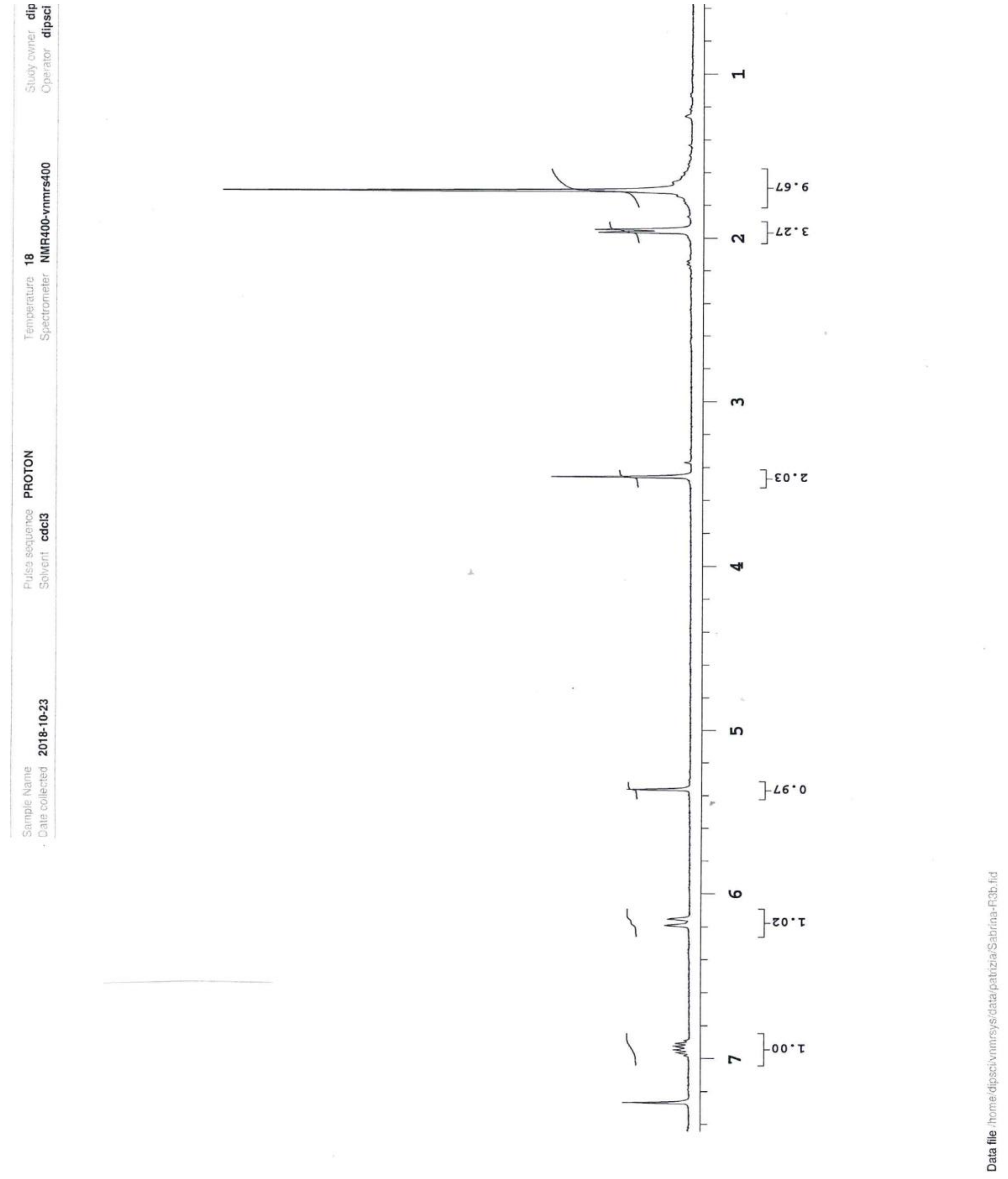

Figure S6. ^{13}CNMR spectrum of compound **6a**

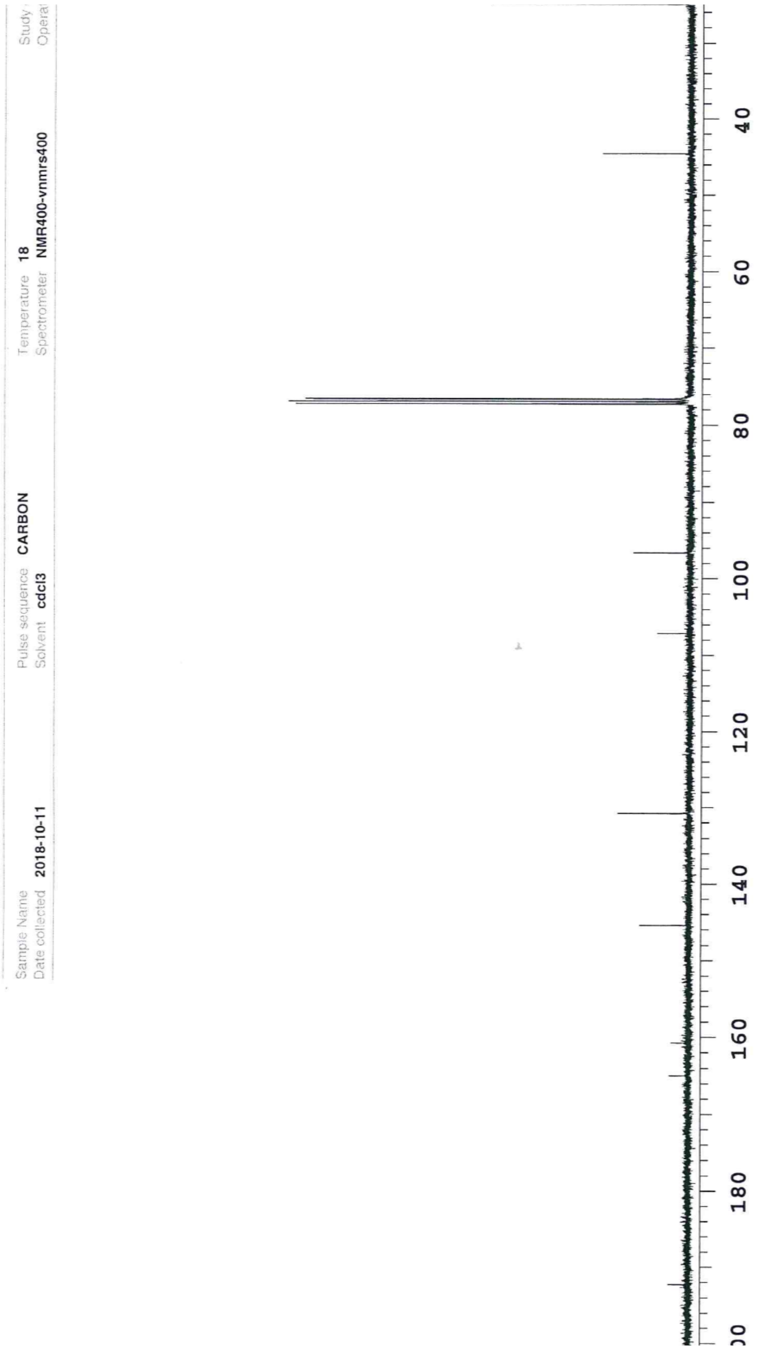

Figure S7. ¹HNMR spectrum of compound **7a**

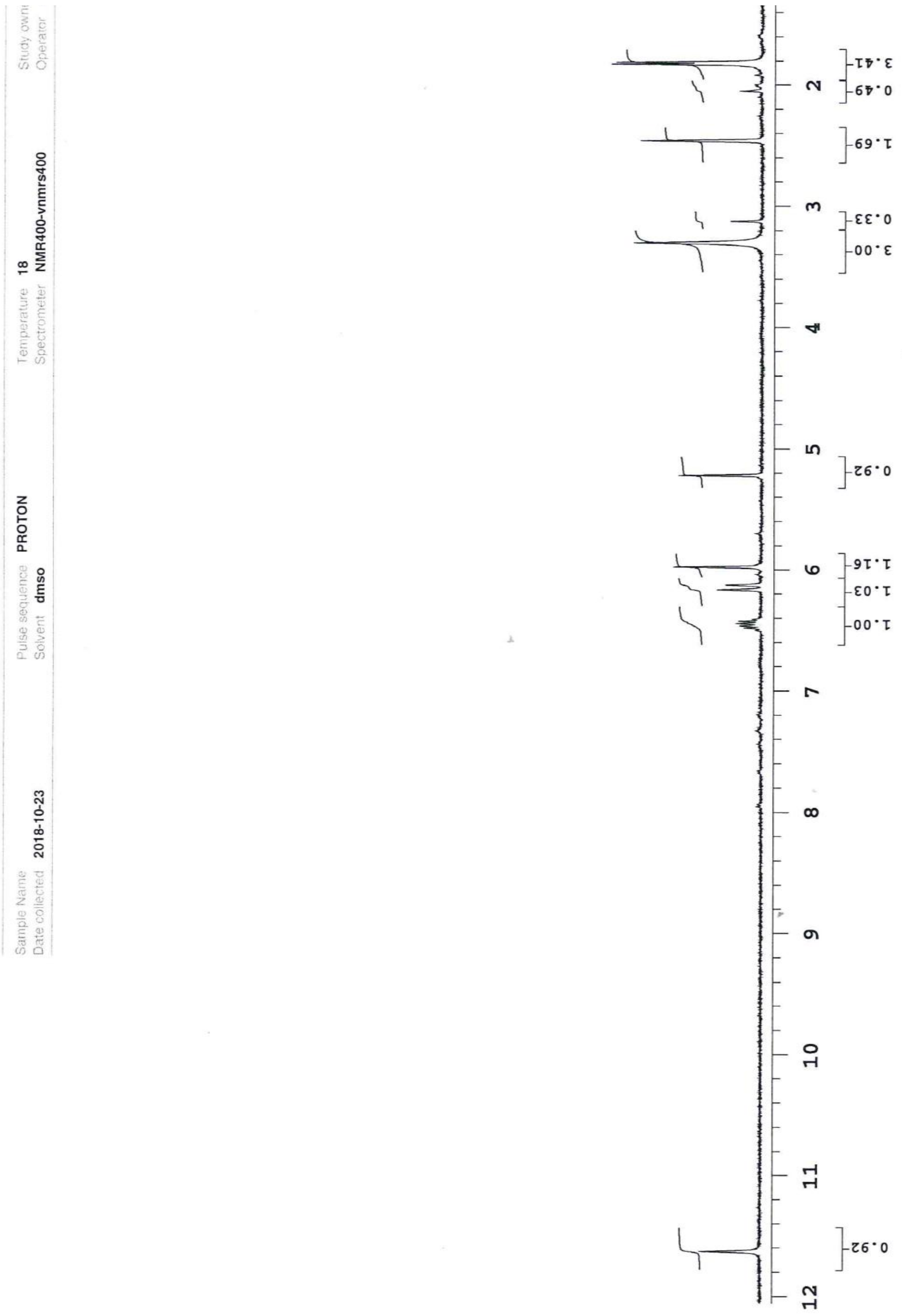

Figure S8. ^{13}CNMR spectrum of compound **7a**

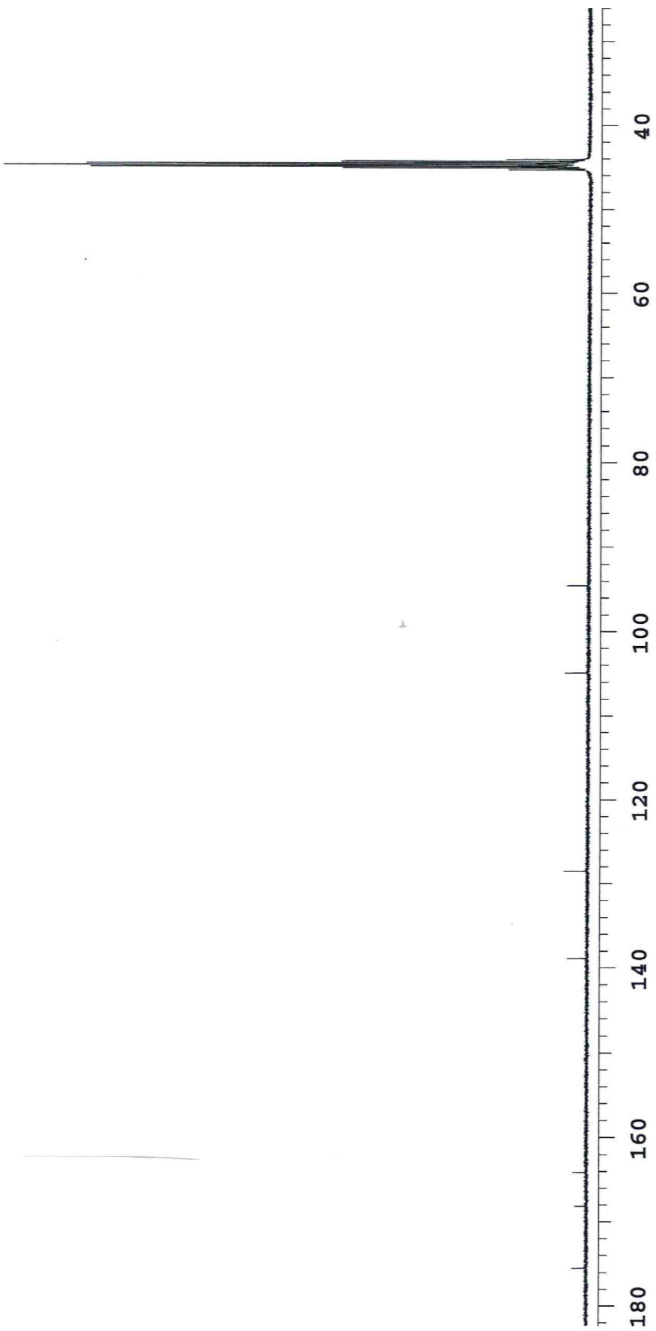

Figure S9. ¹HNMR spectrum of compound **2**

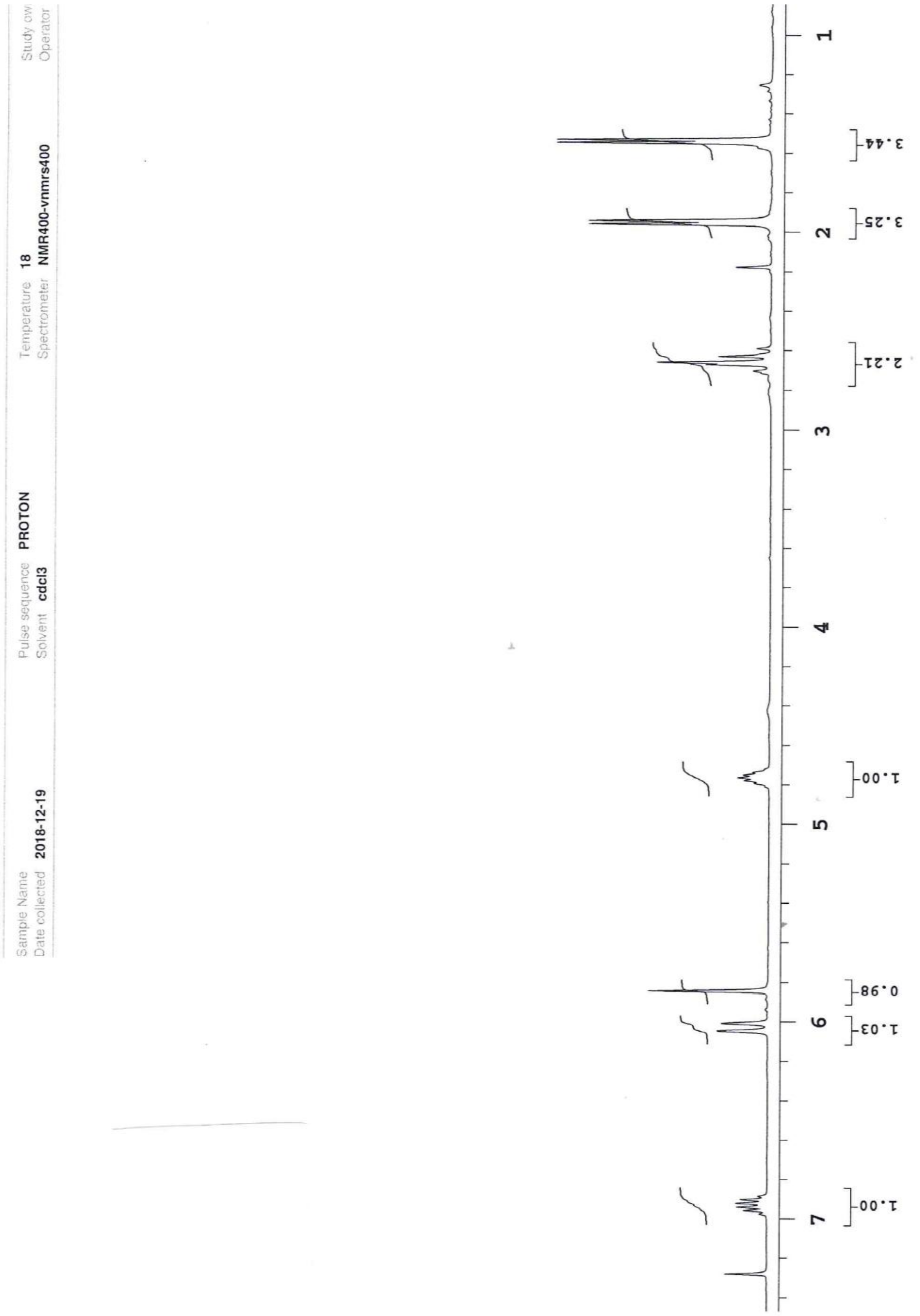

Figure S10. ^{13}CNMR spectrum of compound **2**

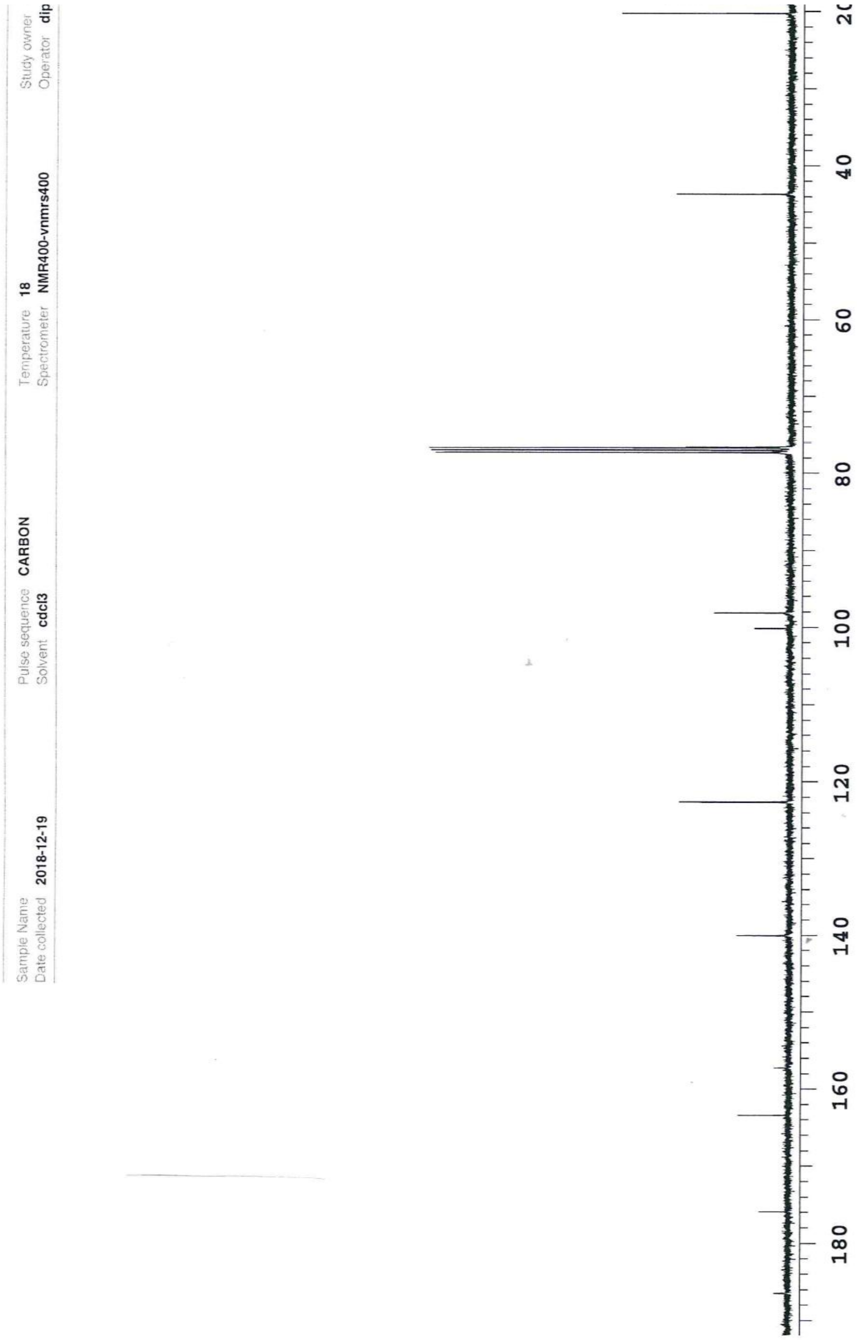

Figure S11. ^{1}HNMR spectrum of compound **9a/9b**

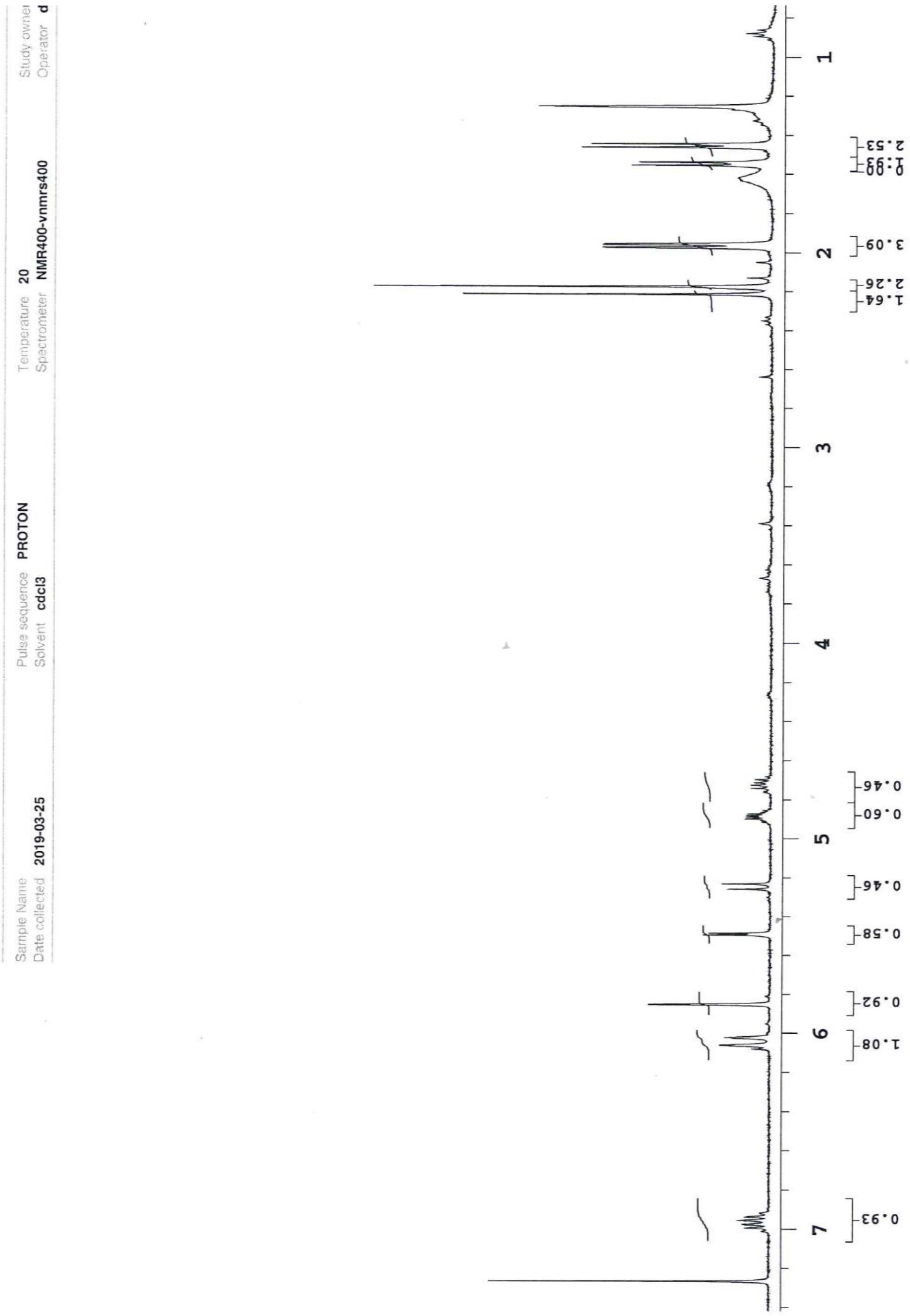

Figure S12. ^{1}HNMR spectrum of compound **10**

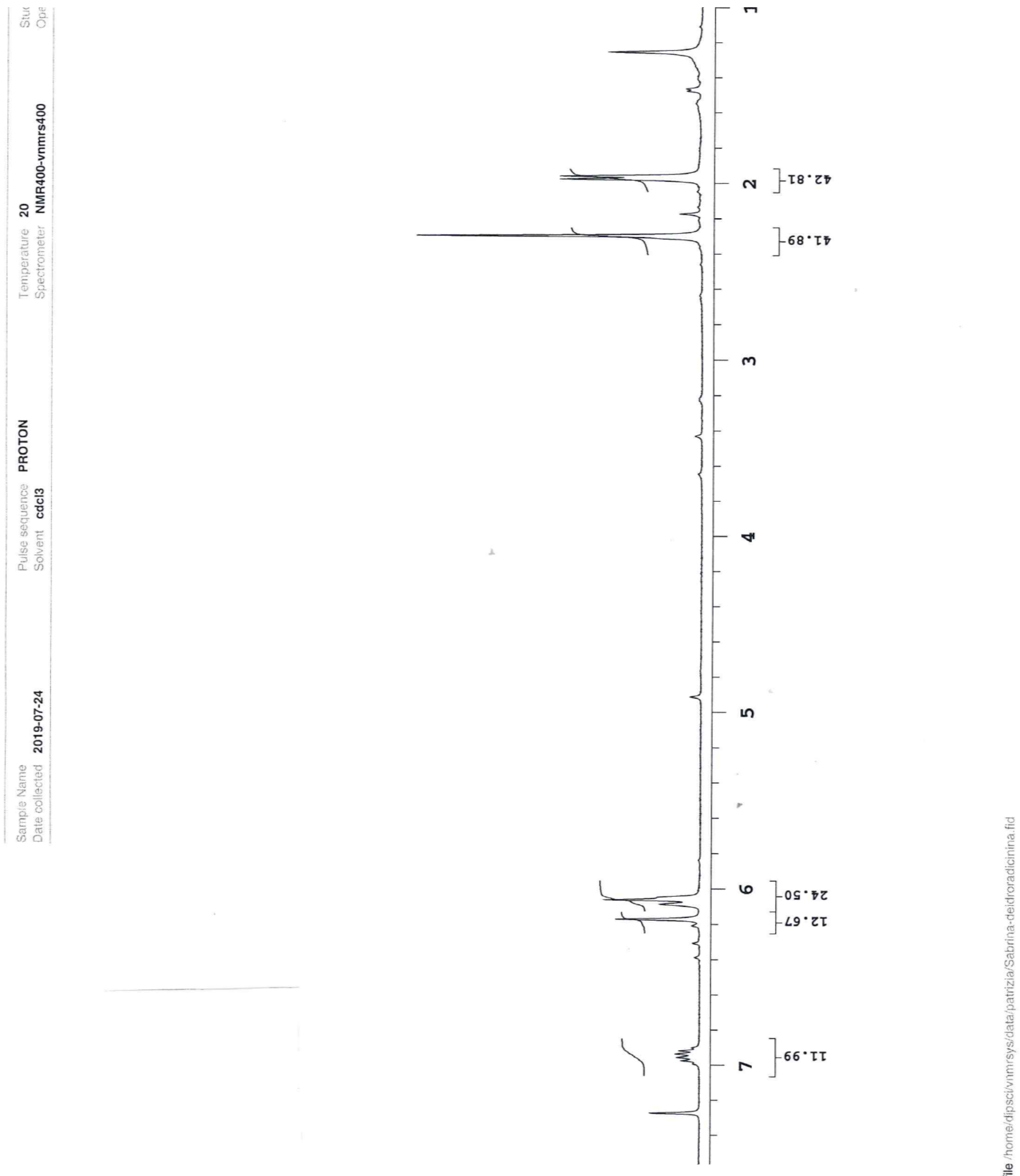

Figure S13. ^{13}CNMR spectrum of compound **10**

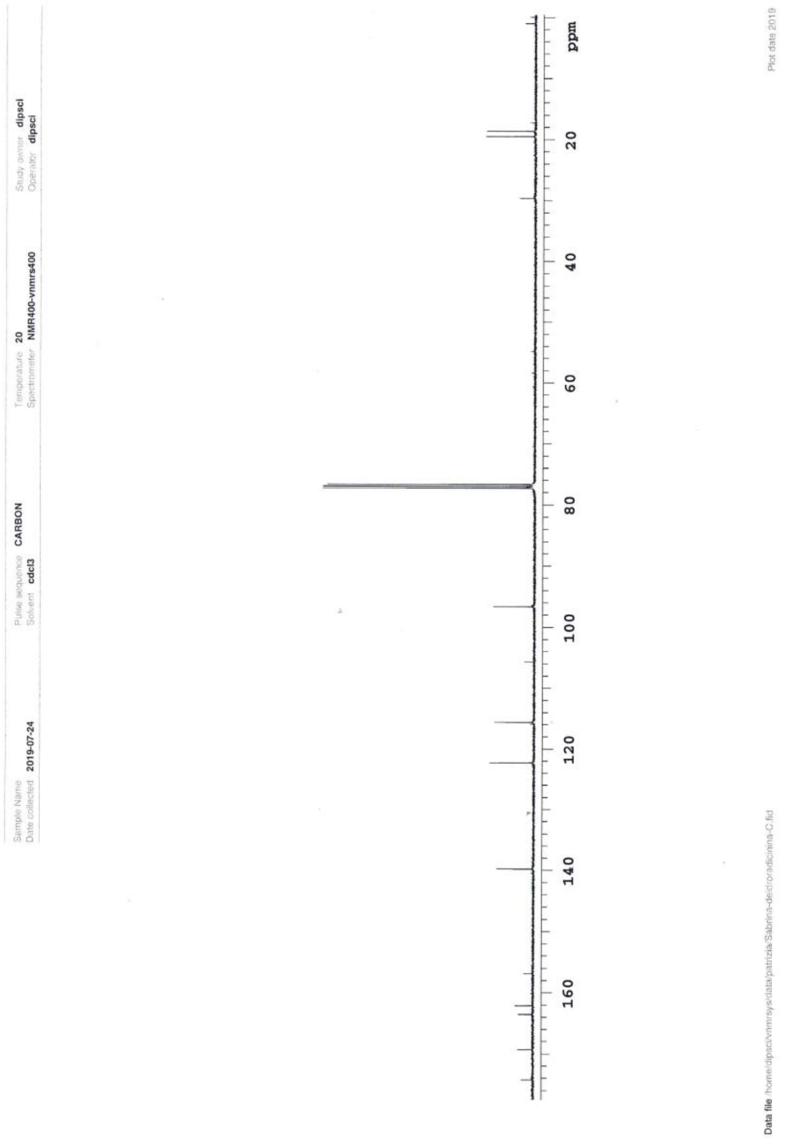

Figure S14. ^{1}HNMR spectrum of compound **5b**

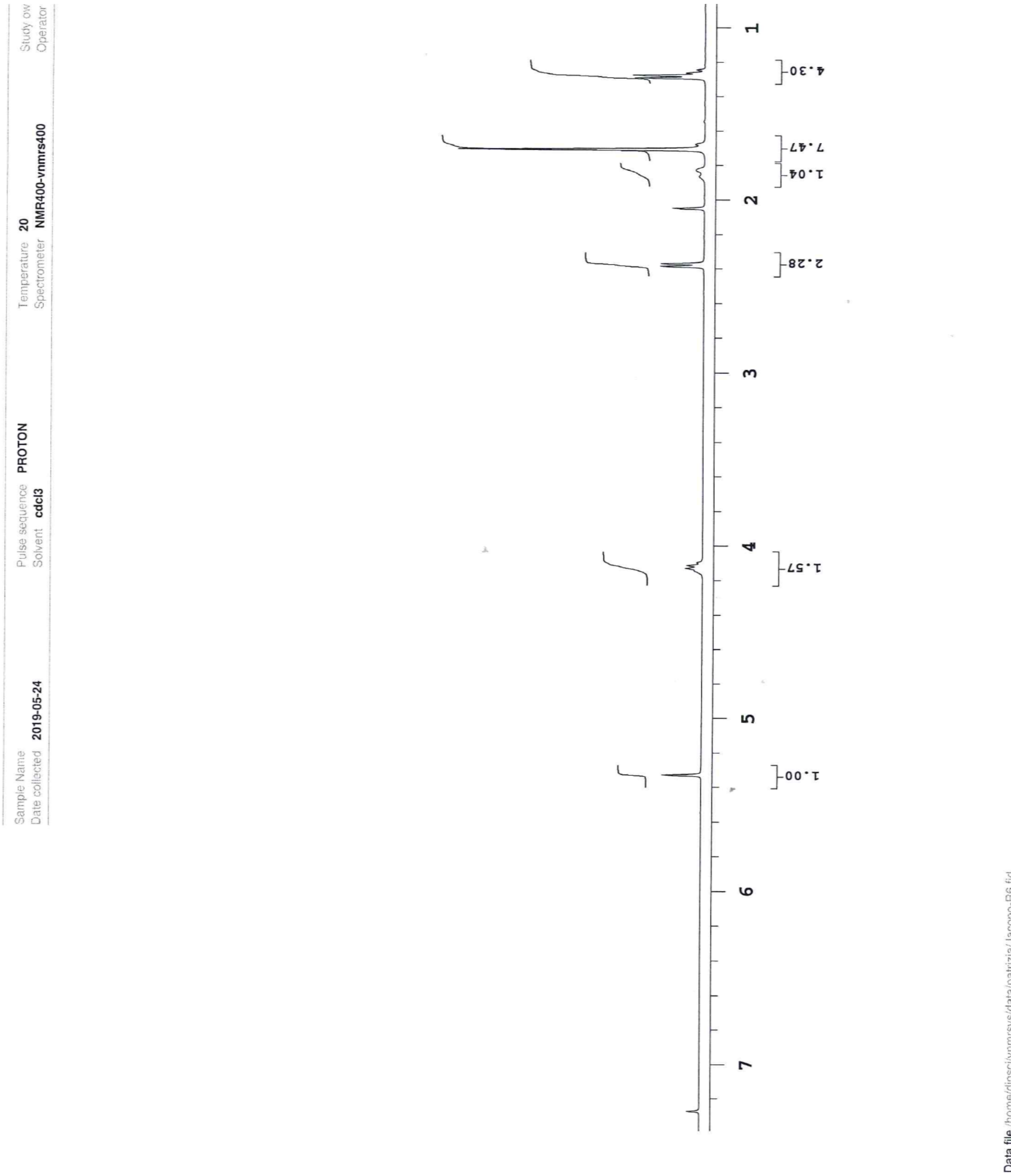

Figure S15. ^{13}CNMR spectrum of compound **5b**

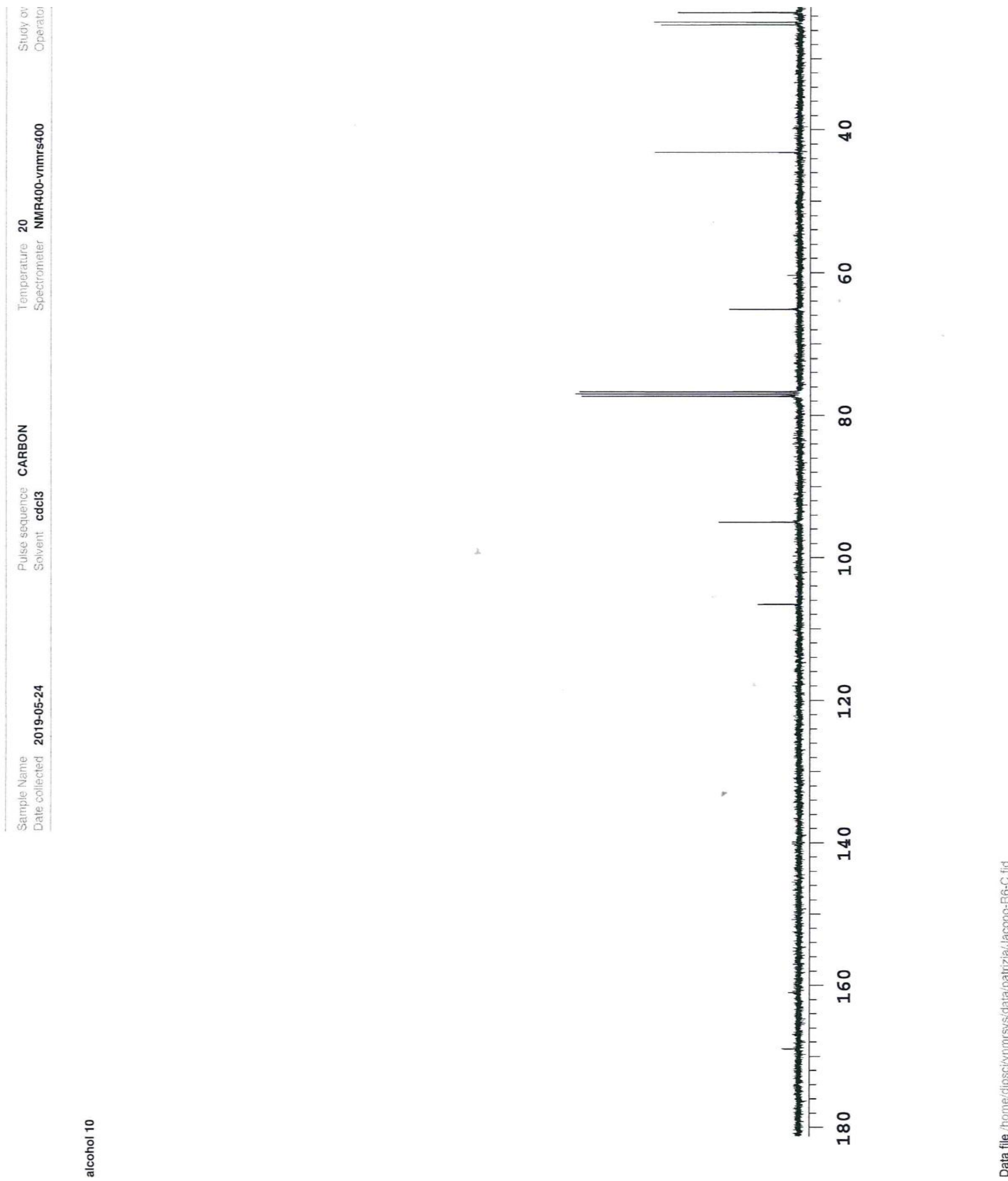

Figure S16. ^{1}HNMR spectrum of compound **6b**

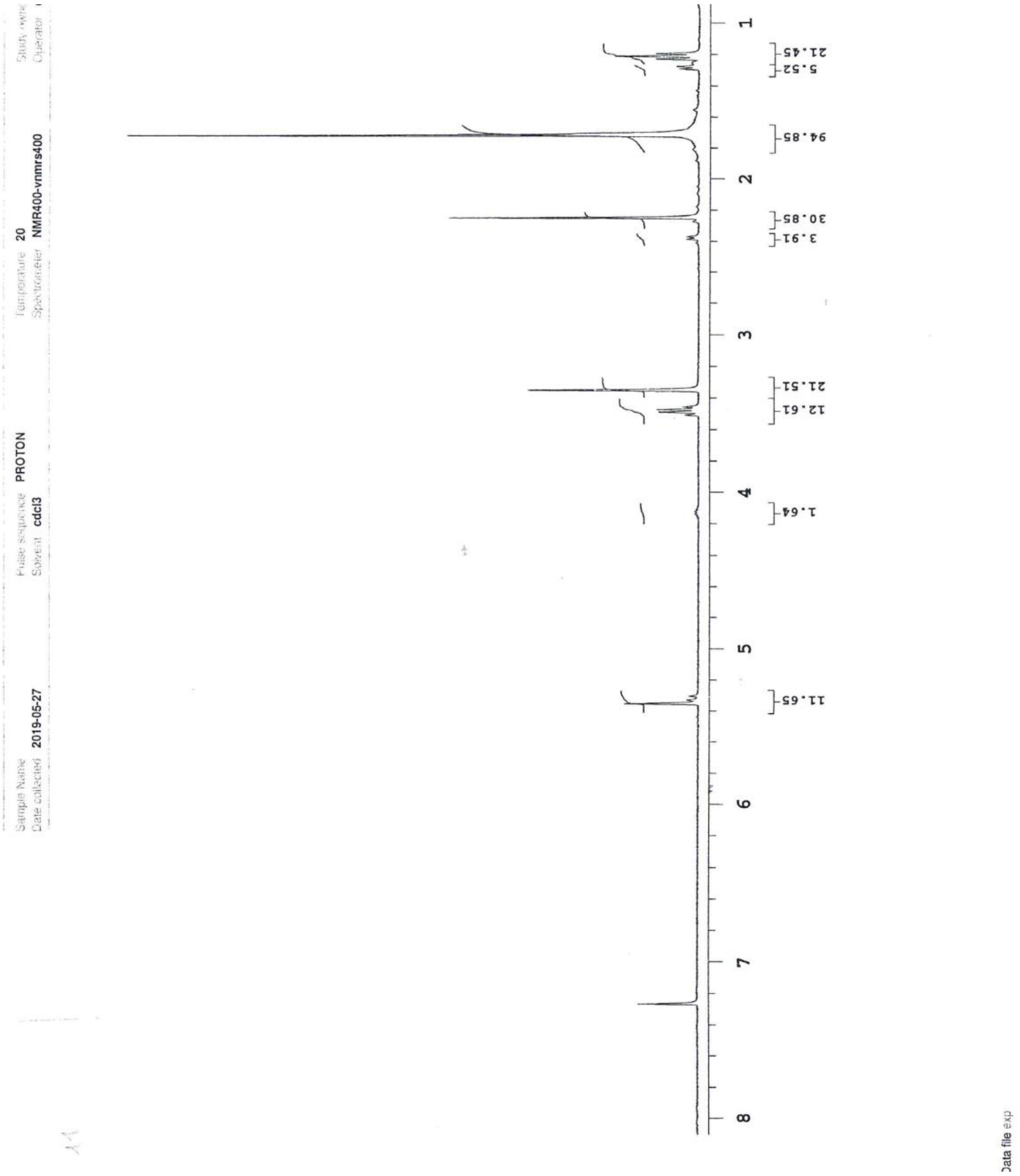

Figure S17. ^{13}CNMR spectrum of compound **6b**

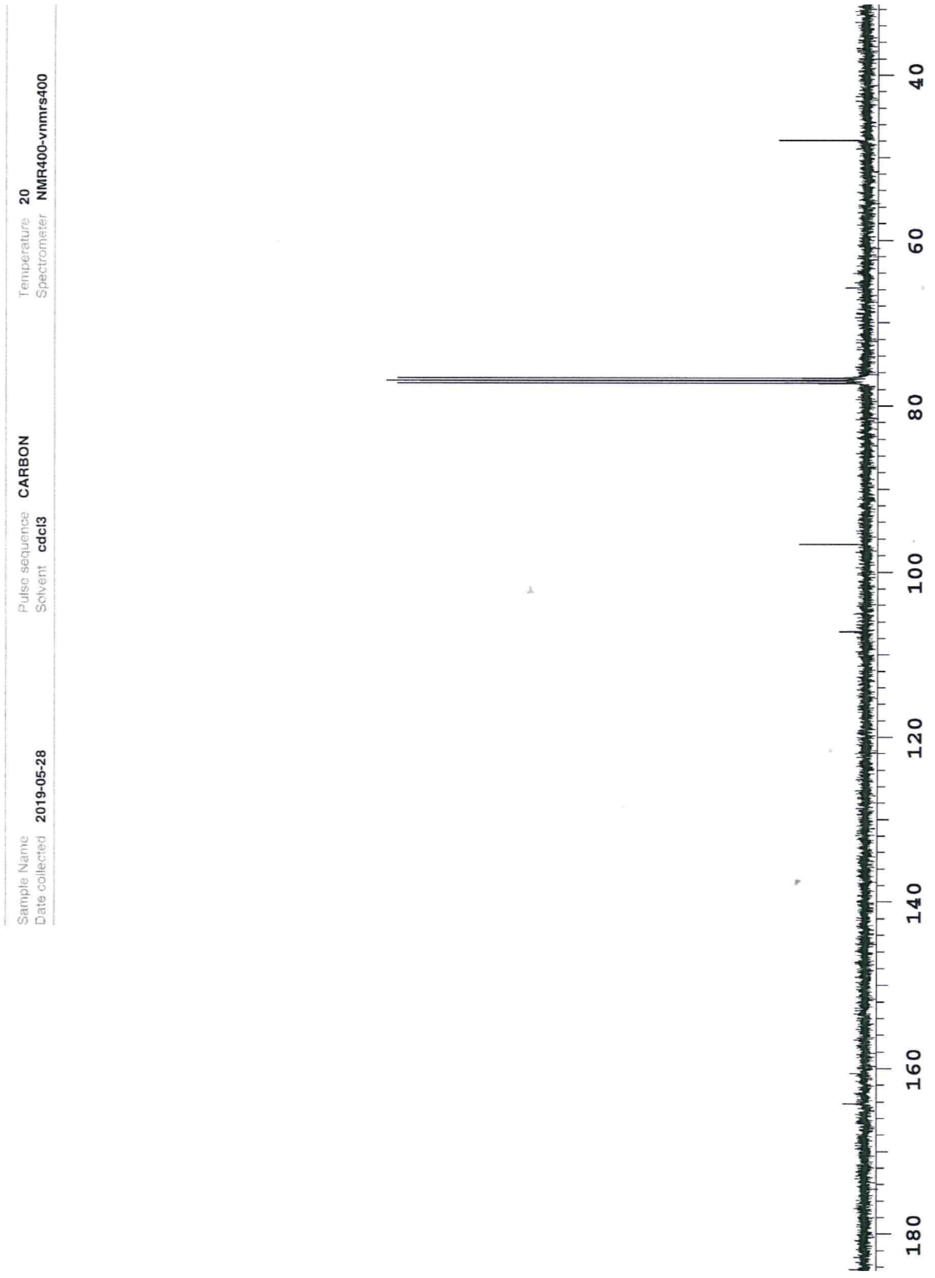

Figure S18. ^{1}HNMR spectrum of compound **7b**

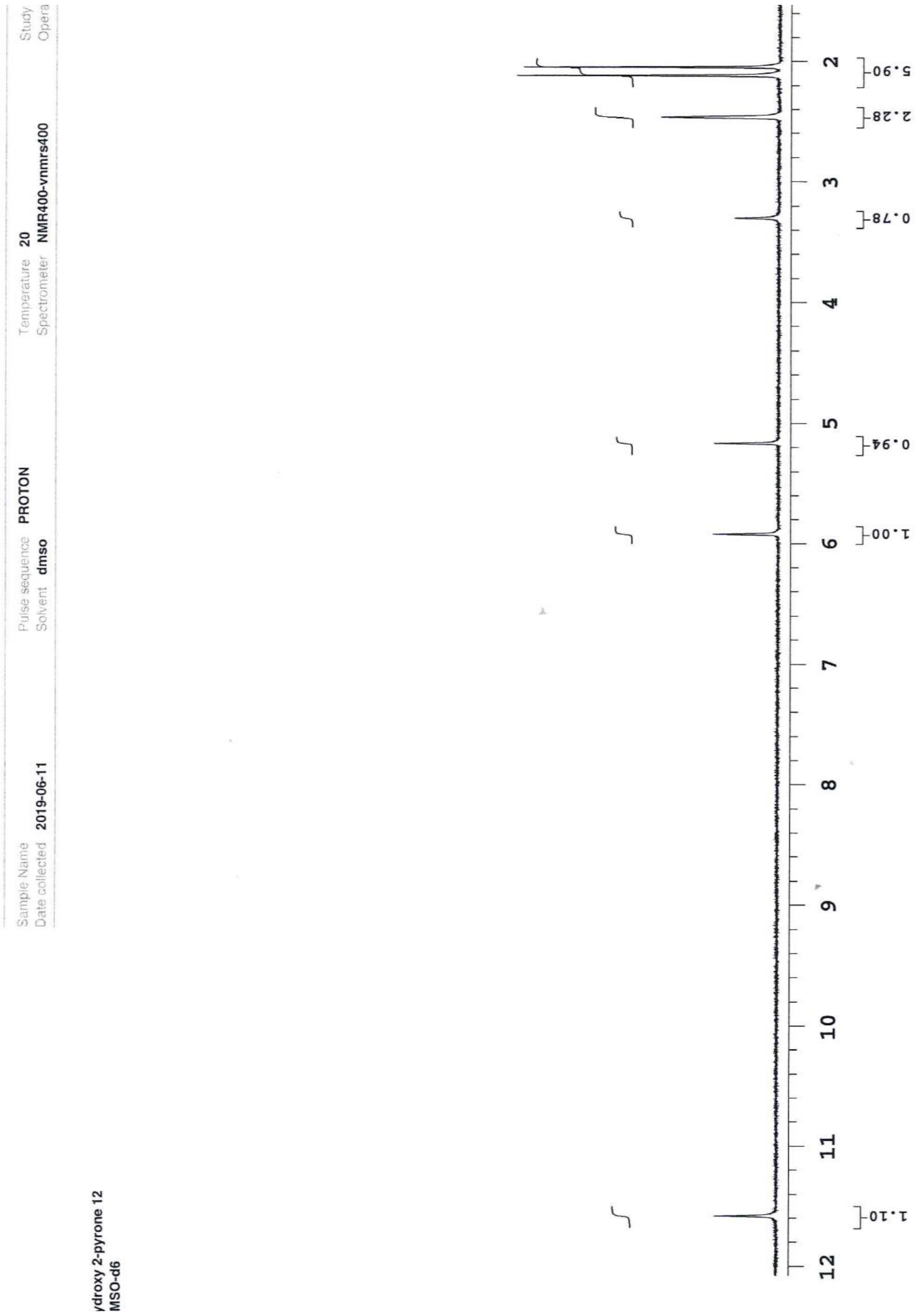

Figure S19. ^{13}CNMR spectrum of compound **7b**

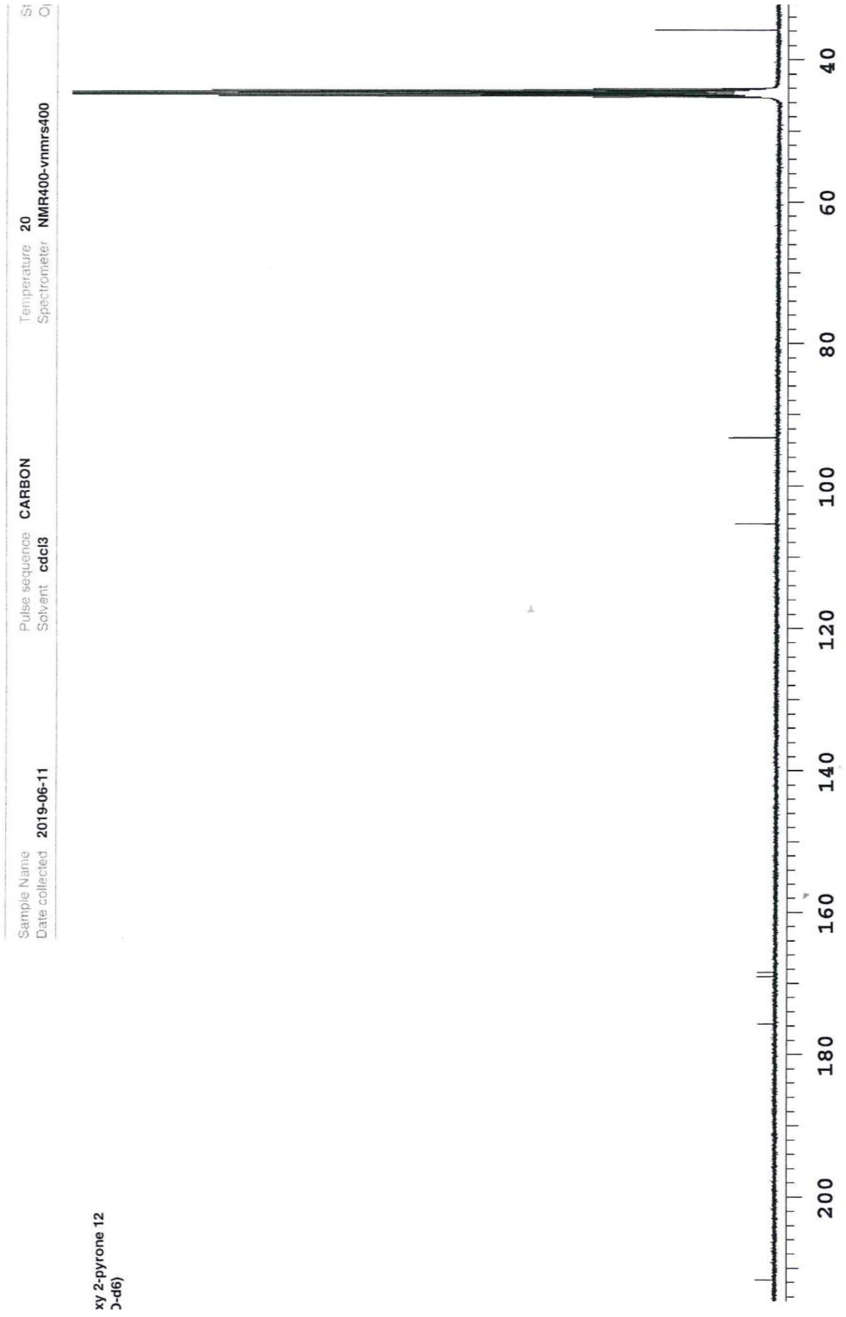

Figure S20. ^{1}HNMR spectrum of compound (±)-**8**

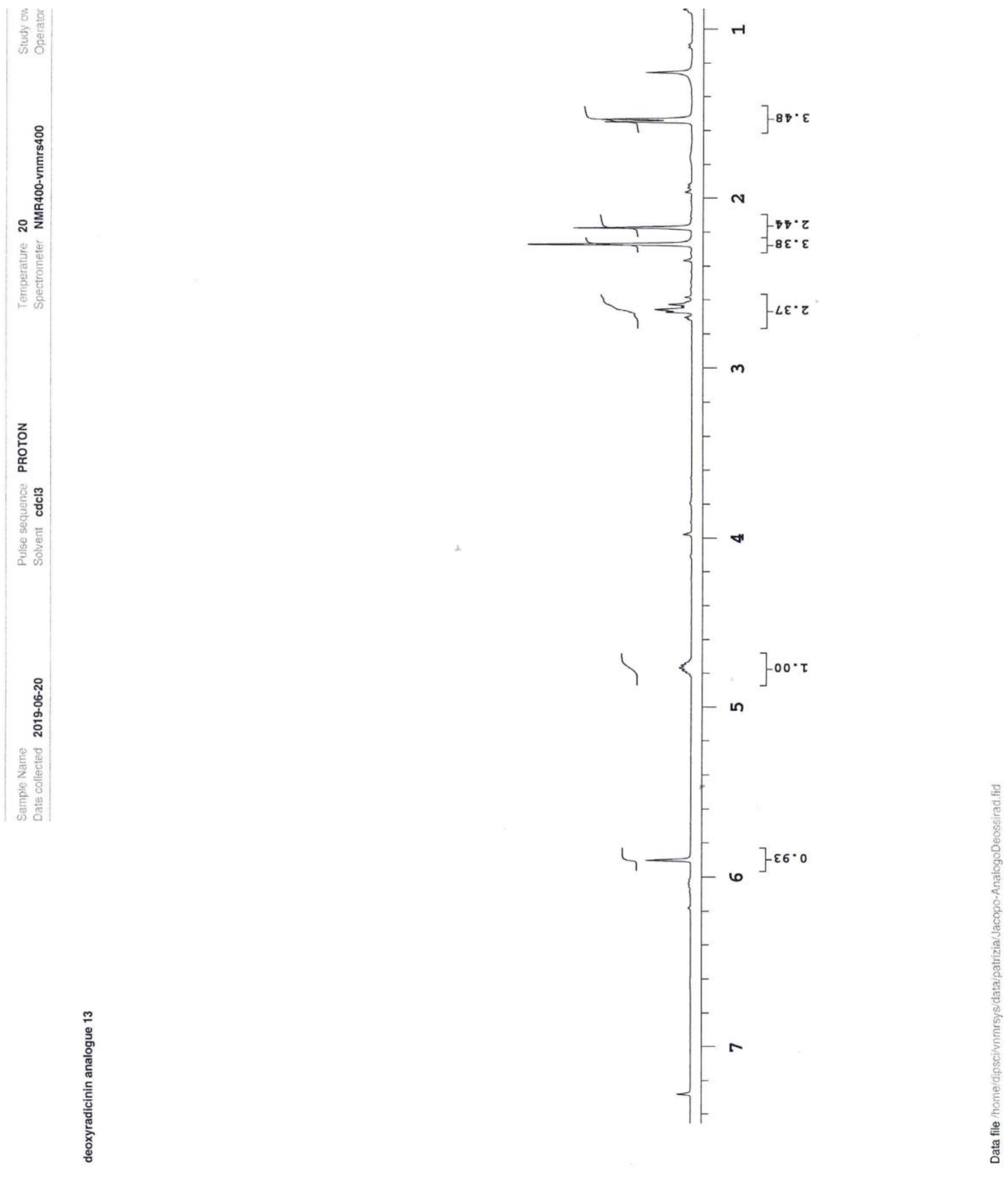

Figure S21. ^{13}CNMR spectrum of compound (±)-**8**

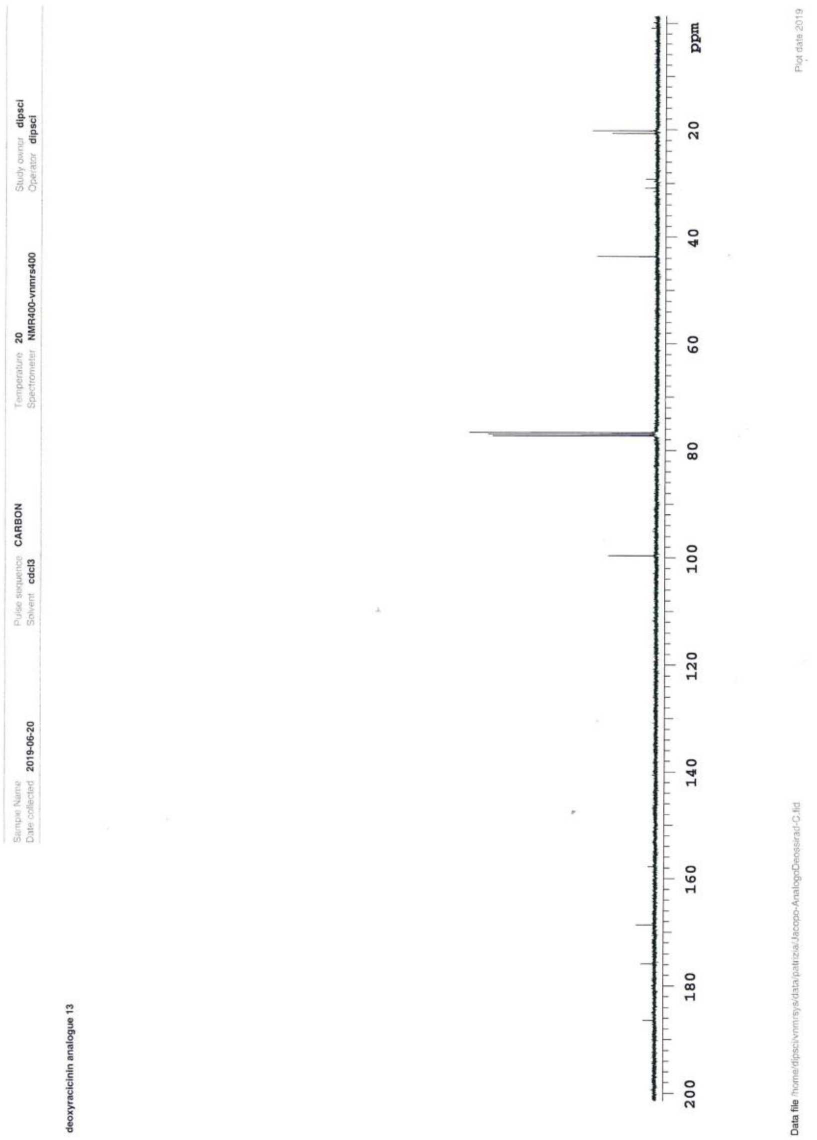

Supplement: Supplementary file 1 [file molecules-24-03193-s001.pdf]
